# Explainable AI in Cancer Imaging: Scoping Review of Methods, Modalities, and Clinical Integration

**DOI:** 10.2196/80645

**Published:** 2026-05-20

**Authors:** Dimitris Fotopoulos, Ioannis Ladakis, Dimitrios Filos, Pedro A Moreno-Sánchez, Mark van Gils, Ioanna Chouvarda

**Affiliations:** 1 Laboratory of Computing, Medical Informatics and Biomedical - Imaging Technologies, School of Medicine, Aristotle University of Thessaloniki, AUTh Campus, Thessaloniki, 54124, Greece, 30 2310999272; 2 Faculty of Medicine and Health Technology, Tampere University, Tampere, Finland; 3 Basque Research & Technology Alliance (BRTA), TECNALIA, Derio, Basque Country, Spain

**Keywords:** cancer imaging, explainable AI, machine learning, medical imaging, trustworthiness

## Abstract

**Background:**

Explainable artificial intelligence (xAI) is increasingly used in medical imaging to enhance transparency, clinical interpretability, and trust in artificial intelligence (AI)–assisted diagnostics, particularly in oncology. Evidence on how explainability is implemented, validated, and reported in cancer imaging remains fragmented.

**Objective:**

This scoping review aimed to systematically map research applying xAI methods to radiologic cancer imaging, summarize methodological and clinical trends, and identify persistent gaps in validation and integration.

**Methods:**

We conducted a structured search of PubMed and Scopus (final search executed on October 20, 2025), covering studies published from 2017 to December 2024. Eligible peer-reviewed articles using machine learning or deep learning were analyzed with a focus on xAI components. Data from 371 studies were extracted into predefined categories covering cancer type, imaging modality, AI model, xAI method, terminology, validation, code availability, and decision support system integration.

**Results:**

Studies focused primarily on breast (112/371, 30.2%), lung (87/371, 23.5%), and brain (56/371, 15.1%) cancers, with prostate, thyroid, and liver cancers also represented. The primary imaging modalities were computed tomography (139/371, 37.5%) and magnetic resonance imaging (104/371, 28%). Deep learning was used in 70.1% (260/371) of studies, classical machine learning in 18.1% (67/371), hybrid pipeline methods for 10% (37/371), and emerging concept-, prototype-, or causal-based approaches accounted for 1.9% (7/371) of studies. Post hoc xAI methods were dominant (305/371, 82.2%), with visualization (163/371, 53.4%), and feature relevance (111/371, 36.4%) as the most common subcategories. Hybrid post hoc or inherent approaches comprised 12.1% (45/371) and intrinsically interpretable methods 5.7% (21/371). Data sources were mostly public (149/371, 40.2%) or mixed (100/371, 26.9%); 22.9% (85/371) used private institutional datasets, and 7.8% (29/371) did not report data sources. Among validated studies, expert or user-based validation was most common (104/193, 53.9%), followed by mixed methods (74/193, 38.3%), while quantitative metrics (10/193, 5.2%) and clinical knowledge–based (8/193, 4.1%) validation remained rare. Only 17.5% (65/371) of studies provided code and 12.1% (45/371) reported decision support system integration, with few achieving actual clinical deployment.

**Conclusions:**

This scoping review maps xAI implementation across multiple cancer imaging modalities, revealing methodological inconsistency and insufficient validation. Most research emphasizes visualization over quantitative interpretability, and few models are reproducible or clinically implemented. These findings provide an evidence base for researchers, clinicians, and regulators to prioritize standardization of xAI reporting, quantitative validation, and user-centered frameworks to advance trustworthy AI in oncology imaging.

## Introduction

### Background on Cancer and Artificial Intelligence

Cancer remains one of the leading causes of mortality worldwide, with lung, colorectal, breast, prostate, liver, as well as leukemia among the most prevalent and costly forms [1,2]. Artificial intelligence (AI)–based systems can improve diagnostic accuracy, reduce analysis time, and identify patterns that might be overlooked by human experts. Such advances indicate a future transformation in the delivery of cancer care [3,4].

A study shows that using deep learning (DL) architecture and radiomics from routine computed tomography (CT) scans can predict clinically significant tumor characteristics, such as staging, without invasive procedures, supporting, in this manner, preoperative risk assessment and the development of treatment plans for patients with cancer [5]. This illustrates both AI’s diagnostic potential and a core tension; the use of less intuitive deep representations, along with hand-crafted features to represent tumors, highlights the need for interpretable results and clinical validation to promote trust and the safe, ethical application of AI in cancer imaging.

The rapid development of AI-based systems is approaching a turning point. A systematic review found that more than 900 AI-enabled medical devices have received Food and Drug Administration marketing approval, with 76% (n=717) of them to be radiology devices—yet among those with available documentation, only 5% (33/717) underwent prospective testing, and 8% (56/717) included a human-in-the-loop [6]. Despite this, it has been argued that even technologically sound and highly accurate AI systems may still face limited clinical adoption if they cannot demonstrate how they reach their conclusions [7]. The recent EU Artificial Intelligence Act (AI Act) of 2024 reflects this concern, classifying AI-based systems in digital medical products intended for cancer diagnosis as “high-risk.” As a result, predictive performance alone will be insufficient, and providers will be required to establish a lifecycle risk management system and adhere to requirements for transparency, human oversight, and robustness [8,9]. In other words, the operation of a high-risk system must be sufficiently transparent so that the deployer can interpret the system’s output and use it appropriately, with clear user instructions and oversight mechanisms. Whether the current generation of AI-based systems—many of which operate as opaque systems—can satisfy these transparency requirements remains an open challenge [10].

### Role of Explainable AI in Clinical Practice

Modern clinical practice emphasizes “human-in-the-loop” collaboration, where AI-based systems augment rather than replace medical expertise, supporting clinical decision-making [11].

However, implementing these AI systems in hospitals and clinics is still in its early stages. The primary challenge stems from the black-box nature of AI models (ie, the reasoning process is hidden), combined with cancer’s complexity as a disease, and the heterogeneity of data [12,13]. This lack of transparency—the difficulty in understanding how AI models reach their conclusions—hinders trust [14] and limits adoption of these AI-based systems in clinical practice [15,16].

Explainable artificial intelligence (xAI) is being developed to address these challenges by making AI decision-making processes transparent and understandable to humans—a quality known as interpretability. Studies demonstrate that this interpretability is essential for establishing clinical confidence and accountability, as well as for integrating AI into clinical practice [17].

However, achieving this interpretability in real clinical settings presents practical challenges. Real-world use of AI in radiology shows that clinical adoption depends on more than just accuracy—it requires workflow integration, staff training, and validation in specific clinical settings to build trust among clinicians and radiologists [18]. AI in cancer imaging encounters similar challenges and requires systems that medical experts can understand and integrate into their workflows to support safe adoption.

xAI implementation in oncology is being pursued through various technical approaches, ranging from visualization tools to advanced computational methods. For example, recent research has developed AI-based systems that convert clinical data into visual formats that the AI can analyze for patterns related to patient outcomes [19]. These systems then generate visual maps highlighting which factors influenced their predictions, allowing clinicians to assess the system’s reasoning. While these approaches show promise for improving clinician acceptance of AI-based systems, extensive validation in clinical settings is needed before widespread adoption.

### Research Gap and Significance of the Review

While several reviews have examined xAI in health care and oncology, most of them focus on specific imaging modalities [20,21] or isolated aspects of interpretability [22,23]. A critical gap remains; we lack a comprehensive understanding of how xAI methods are actually being implemented, validated, and prepared for clinical use, specifically in cancer imaging.

A recent analysis of radiology AI-based systems reveals a concerning pattern [24]. Although xAI methods are increasingly included in DL-based computer-aided diagnosis, they remain mainly limited to simple visualization methods. More importantly, these explanations are rarely evaluated quantitatively or validated with actual clinicians [25]. This may be due to the lack of standardized frameworks for evaluating the quality of explanations generated, which presents an additional challenge for researchers attempting to evaluate and compare various methodologies in terms of their ability to produce clinically acceptable results [26].

Furthermore, a comparative evaluation across different imaging modalities and clinical applications has also shown that the accuracy and stability of explanations provided by each methodology can differ substantially depending on the application, and several methodologies underperform significantly with even slight changes to input data [27]. This suggests that selecting an appropriate xAI method for a particular clinical use case requires careful consideration of the task and the type of medical images, to choose a method that provides the most robust results.

A study examining technical and clinical perspectives on AI validation identified a misalignment regarding what is necessary for informed decision-making, comparing the technical transparency features integrated by developers with the actual informational requirements of clinicians [28]. If there is a chasm between the information clinicians need to make informed decisions and the information provided by system developers due to documentation required for high-risk regulatory classification, it raises the question of whether current systems are developed to meet clinician needs or simply to fulfill documentation requirements for regulatory purposes.

Taken together, these concerns prompt a fundamental question—do these xAI methods genuinely support clinical decision-making, or do they merely provide technical justification after the fact [29]? Adding to this concern, limited code and data sharing practices hinder reproducibility, making it difficult to independently verify explainability claims or assess real-world clinical utility [30]. Without systematic validation and clinical evaluation, xAI risks becoming a “checkbox” feature rather than a tool that improves patient care.

This scoping review addresses these gaps by systematically examining how xAI methods are implemented, validated, and prepared for clinical use in cancer imaging. Large-scale research initiatives, such as the European Cancer Imaging Initiative [31], are developing infrastructures to support AI training and evaluation at scale, making it increasingly important to understand how explainability is integrated into these systems.

The analysis will reveal whether technical capabilities in explainability are matched by rigorous validation and real-world integration—identifying the disconnect between what xAI methods can do and whether they actually improve clinical decision-making. By mapping the current landscape, this review provides an evidence base for improving xAI research and practice. The findings will help researchers identify methodological gaps, guide clinicians in understanding which explanation methods have undergone rigorous evaluation, and inform institutions and regulators about the current readiness of xAI-enabled cancer imaging systems for clinical deployment.

### Objectives of This Review

To address these gaps, this scoping review examines how explainability is implemented, validated, and reported across cancer imaging AI research. Unlike previous reviews that focus on single imaging modalities or isolated technical aspects, this review takes a comprehensive approach, covering multiple imaging modalities and both technical and clinical dimensions of xAI.

Specifically, this scoping review aims to address the following research questions:

1. How is explainability used in cancer imaging AI (post hoc vs intrinsically interpretable and model-specific vs model-agnostic), and how has this distribution evolved until 2024?
2. To what extent are explainability methods validated and reported in a standardized manner through user studies, expert evaluation, quantitative metrics, or domain-specific assessment?
3. What is the current state of reproducibility and clinical readiness of xAI in cancer imaging, including dataset availability, code sharing, and integration into decision support systems (DSS), and which barriers hinder the real-world adoption the most?

## Methods

### Framework and Reporting Standards

The conduction of the research and the writing of this scoping review were done according to the PRISMA-ScR (Preferred Reporting Items for Systematic Reviews and Meta-Analyses extension for Scoping Reviews) framework [32]. In addition, the reporting of the search strategy follows the PRISMA-S (Preferred Reporting Items for Systematic Reviews and Meta-Analyses Search Extension) guidelines to ensure transparency in documenting information sources, full search strategies, search limits, and deduplication procedures [33]. The completed PRISMA-ScR checklist is provided in Checklist 1.

### Information Sources

To comprehensively review the literature, we developed specific search queries for PubMed and Scopus. The database interfaces used were PubMed (NCBI) and Scopus (Elsevier), as recommended for transparent reporting under PRISMA-S guidelines. PubMed was selected for its comprehensive coverage of biomedical literature, making it ideal for capturing studies on AI in medicine and cancer. Scopus was chosen due to its broad coverage of scientific literature across multiple domains, including engineering and medicine. Scopus also indexes many journals not listed in Web of Science. We prioritized peer-reviewed journal articles and excluded earlier materials and nonjournal categories, such as conference abstracts or book reviews.

### Search Strategy

To identify relevant literature, we created comprehensive search queries targeting studies on xAI in the context of cancer treatment. The search was conducted in PubMed and Scopus using keywords and MeSH (Medical Subject Headings) on the topics of explainability, interpretability, accountability, trust, AI, and cancer. These terms were selected following an initial analysis of key publications, which allowed us to identify the most commonly used concepts and terminology related to explainability in AI research.

Search reporting elements—including database platforms, complete search strings, search dates, applied limits, and the deduplication approach—are documented in accordance with PRISMA-S recommendations. Full search syntaxes for each database are provided in Multimedia Appendix 1.

The PubMed query combined free text and MeSH terms with Boolean operators and field markers (eg, [tiab] and [MeSH Terms]). The Scopus query was structured with TITLE-ABS-KEY operators to search titles, abstracts, and keywords. In Scopus, certain terms (eg, *accountab* and trust*) were restricted to the TITLE-ABS fields to minimize retrieval of nonrelevant records that often arise when these terms appear only in keywords. Both queries excluded reviews, meta-analyses, and non–English language articles. Consistent with PRISMA-S recommendations, we documented all applied limits, including restriction to English-language records and the exclusion of reviews, meta-analyses, systematic reviews, and non–peer-reviewed materials, such as conference abstracts or book chapters.

The initial search was executed in January 2024, covering records from 2017 to 2023. No restrictions were placed on data modalities, ensuring broad inclusion of studies from different subfields of AI research related to cancer. Following peer review, the search was updated on October 20, 2025, to include studies published through December 2024. Exact search dates for each database are reported in Multimedia Appendix 1. The full query syntaxes for both databases can be found in Multimedia Appendix 1.

Duplicate records across databases were removed using a custom Python script that identified duplicates based on matching DOI fields, followed by manual verification to ensure accuracy.

### Eligibility Criteria

Medical imaging modalities such as CT, positron emission tomography (PET), x-ray, mammography, magnetic resonance imaging (MRI), and ultrasound were included in the review because of their central role in cancer diagnosis, treatment planning, and frequent use in AI research. These radiologic imaging techniques provide noninvasive, clinically relevant information, and are widely used for monitoring cancer progression. In contrast, imaging modalities primarily used in other biomedical fields, such as histopathology, immunofluorescence, and dermoscopy, were excluded to maintain focus on radiologic data.

We included articles published from 2017 to December 2024. Inclusion criteria were:

1. Introduces or evaluates an AI system applied to cancer care with a focus on explainability, interpretability, or trustworthiness;
2. Published in peer-reviewed journals or as full, peer-reviewed conference papers presented at conferences (conference abstracts were excluded);
3. Written in English;
4. Not a review, opinion, editorial, commentary, or meta-analysis article.

Duplicates were removed. Studies that only mentioned explainability or interpretability superficially without methodological detail were also excluded. The search strategy and its reporting adhere to PRISMA-S guidance, ensuring transparent documentation of all search components, including database interfaces, search terms, restrictions, and handling of duplicates.

### Data Extraction and Information Categories

The data extracted are organized into the following categories:

- Study identification: title, authors, year, and source of publication.
- Study characteristics: cancer type, primary objective, dataset description, and sample size.
- Data and methods: data modalities, preprocessing steps, features used, feature selection, and machine learning (ML) or DL algorithms.
- Clinical aspects: validation approach and clinical performance metrics.
- Explainability aspects: xAI methods used, their purpose, terminology, and user involvement.
- Results and limitations: key findings and reported limitations.

The detailed data extraction schema, including all the field names and descriptions, is available in Multimedia Appendix 2.

D Fotopoulos conducted the initial search and deduplication. D Fotopoulos and D Filos screened titles and abstracts. Studies were randomly distributed among the reviewers—D Fotopoulos, D Filos, and IL—and they proceeded to perform the full-text reviews. To ensure consistency across reviewers, PAMS independently reviewed a random sample of 10 studies from each batch of full-text reviews. If substantial discrepancies were observed, PAMS would increase the audit sample; conflicting evaluations were resolved by discussion among all reviewers, ensuring consensus before final inclusion decisions.

### Synthesis of Results

Extracted data were managed in Microsoft Excel and analyzed using descriptive mapping with frequency counts, consistent with Joanna Briggs Institute scoping review methodology. Study characteristics (cancer type, imaging modality, dataset availability, code availability, and integration into a DSS) were coded at the time of extraction using predetermined categories established before the extraction process began. The explainability methods used in each study were categorized according to the framework described in the next section, based on a taxonomy developed by Arrieta et al [34], including the xAI strategy (inherent, post hoc, or hybrid), post hoc method family (text, visual, local, example-based, simplification, or feature relevance), model dependence (model-specific or model-agnostic), and explanation scope (local or global). Categories of validation approaches were not established a priori; however, through an inductive approach during data extraction based on the strategies reported by the included studies, 4 categories (expert or user-based, performance-based, domain or clinical knowledge-based, and mixed methods) emerged. Results will be presented as frequencies and percentages, with temporal trends evaluated when applicable. No meta-analysis or interpretative qualitative synthesis was conducted, consistent with the descriptive mapping goals of this scoping review.

### Background and Theoretical Framework

#### Overview

This section defines the terminology and classification system for xAI methods reviewed in cancer imaging studies. The objectives are (1) to provide a standard way of referring to “interpretability,” “explainability,” and “transparency” in the context of this review; (2) to group xAI techniques commonly reported in studies into reproducible families of methods; and (3) to identify how each study documented its intended application of explainability and whether any evaluations of that explainability were conducted.

The concepts and taxonomic distinctions referenced in this section are based on previous research related to the field (eg, Arrieta et al [34], Holzinger et al [35], and Roscher et al [36]). In other words, our goal in mapping each included study to this established landscape is to use the existing framework for reference.

#### Scope of This Framework

This review analyzed 371 studies (Multimedia Appendix 3) that used various terms to describe “interpretability, “explainability,” and “transparency” for presenting their research findings with a wide range of xAI techniques. For each study included, we mapped its explainability approach onto 4 dimensions. First, we recorded the xAI strategy, distinguishing whether the approach was inherently interpretable, post hoc, or a hybrid of the two. Second, for studies that used post hoc xAI, we assigned one or more post hoc method categories, drawing on the families defined by Arrieta et al [34], which include text explanations, visual explanations, local explanations, explanations by example, explanations by simplification, and feature relevance explanations. Third, we documented the model dependence of each technique, classifying methods as either model-specific or model-agnostic. Finally, we captured the scope of the explanation, noting whether it was intended to be local (instance-level), global (model- or dataset-level), or a combination of both.

#### Key Concepts

Explainability and interpretability are 2 terms that can be found used interchangeably in some cases, although some literature suggests they should be distinguished, as they convey different meanings [37,38]. Both support transparency, which refers to clarity regarding the model’s structure, data, and assumptions, a cornerstone of trustworthy AI [39]. We use the following working definitions to prevent ambiguity throughout this review.

Interpretability refers to an intrinsic (passive) property of a model: the extent to which a human can directly understand how inputs are mapped to outputs without requiring an additional explanatory mechanism. Roscher et al [36] describe interpretability as the inherent comprehensibility of a model. Linear regressions or shallow decision trees (DTs) are interpretable by design because their parameters map directly to explicit features, and Arrieta et al [34] relate this notion to transparency.

We use explainability to refer to an active property: the ability of a system to generate reasons, evidence, or visual and linguistic justifications for its outputs in a form intended to be understandable to a target audience. Explainability may be inherent to the architecture (eg, a model that surfaces explicit prototypes or attention maps as part of its forward pass), or it may be added after training via post hoc analysis of an otherwise opaque model.

Transparency involves openness in model structures, data, and design assumptions. Transparency—informally the opposite of opacity—varies by context and user and can be analyzed across 5 dimensions—human involvement, data, the model, inferencing, and algorithmic presence [40]. Also, according to Arrieta et al [34], which is the one that guides this review, can be divided into 3 levels:

- Simulatability: a human can mentally reproduce the model’s reasoning,
- Decomposability: each component (inputs, parameters, and computations) is interpretable, and
- Algorithmic transparency: the clarity of the learning procedure itself. Linear and logistic regression models typically meet all 3.

These distinctions matter because the terms “interpretable,” “explainable,” and “transparent” are not used consistently. In several instances across the literature, authors describe their approach as “transparent” primarily because the underlying model class is simple (eg, a shallow DT or sparse linear model). Other studies provide only post hoc saliency maps for a deep neural network and still refer to the solution as “transparent.”

In our review, we do not assign a transparency rating to each research study to measure the levels of simulatability, decomposability, or algorithmic transparency. Instead, we record (1) which model architecture was used and (2) whether interpretability is claimed to be inherent or is provided through a separate post hoc explanation method. This allows us to report, in the Results section, how often explainability is treated as a built-in design property versus an add-on.

### Taxonomy of xAI Methods

#### Overview

To compare explainability strategies across studies, we group reported methods along these axes widely discussed in xAI research and directly aligned with our extraction fields: xAI *method*, *type of xAI*, and *aim of interpretability or explainability*.

A central distinction in this taxonomy is whether a study uses an interpretable-by-design model, a post hoc explainability technique, or a combination of both. Interpretable-by-design approaches construct the model so that its internal decision process is directly understandable. Examples include linear models built over predefined features, shallow DTs, rule-based systems, and prototype-based architectures in which predictions are explicitly linked to reference exemplars; in these cases, the explanation is embedded within the model itself. Post hoc explainability methods, by contrast, are applied after training to elucidate the behavior of otherwise opaque models, typically through outputs such as saliency maps, pixel or feature importance scores, or simplified local surrogate models. Hybrid approaches appear in studies that use an inherently interpretable model while also incorporating an additional post hoc xAI method.

During extraction, we map each study to one or more of these categories, based on how they describe their approach in *ML or DL method used, type of xAI,* and *xAI method* fields.

#### Model-Specific vs Model-Agnostic Methods

We then classified explainability techniques based on whether they depend on the internal structure of a particular model class. Model-specific methods rely on architecture-specific information, such as gradients, activation maps, attention scores, or layer relevance. Examples include gradient-weighted class activation mapping (Grad-CAM) and its variants for convolutional neural networks, attention-weight visualization in transformer architectures, and layer-wise relevance propagation. Because they incorporate internal model characteristics, these approaches generally cannot be applied unchanged to other model families. In contrast, model-agnostic methods treat the model as a black box and infer explanations by probing input-output behavior. Representative techniques include local interpretable model-agnostic explanations (LIME) [41], Shapley Additive Explanations (SHAP) [42], perturbation-based feature importance, occlusion sensitivity analyses, and locally fitted surrogate models for individual predictions. Since they do not depend on internal model layers, these methods can, in principle, be applied across a wide range of classifiers or regressors, including ensemble architectures.

#### Local vs Global Explanations

We have separated our methods into local explanations (explanations for a single prediction, such as heatmaps of a suspected malignancy region in 1 patient) and global explanations (overview of model performance across the entire dataset, such as top-ranked lists of the most influential features). For each method, we have documented how it describes its own scope as local, global, or both. We note that “local explanation” appears in xAI categorization by Arrieta et al [34], where “local” refers to a family of methods focused on sample-by-sample justification, typically using surrogate models of local data.

#### Families of Explainability Methods

Finally, for studies that use post hoc explainability, we assign each reported method to one or more method families, which follow the taxonomy presented in the study by Arrieta et al [34].

1. *Text explanations:* Methods that generate a verbal, symbolic, or natural language rationale for prediction. In imaging, this includes approaches that describe suspicious morphology (eg, spiculation and enhancement patterns) or provide a rule-like statement in words.
2. *Visual explanations*: Methods that produce visual proofs, intended to show what the model relied on. In the context of medical imaging, this could be a heatmap, an attention map, or activation overlay on the original image series to indicate regions of interest.
3. *Local explanations:* Methods that aim to explain an individual prediction in its local neighborhood, typically by constructing a simplified, human-readable surrogate just for that case (eg, a local linear model or a shallow local DT), or by providing a per-instance strategy.
4. *Explanations by example*: Methods that justify a prediction by retrieving or referencing similar prior cases, exemplars, or learned prototypes (“this lesion is malignant because it resembles these malignant prototypes”). This follows medical experts’ (eg, radiologists) reasoning process.
5. *Explanations by simplification:* Methods that approximate a complex model with a simpler surrogate; for example, extracting a rule set, fitting a shallow DT, or learning a sparse linear model that mimics the original model locally or globally. The goal is to communicate decision logic in a more interpretable form.
6. *Feature relevance explanations:* Methods that assign importance scores to features, pixels, or regions to indicate which inputs most influenced the model’s prediction.

Individual studies may report more than one category (eg, both saliency maps and a local surrogate model). We record all categories. In the Results section, we report the prevalence of each category across cancer types and imaging modalities.

## Results

### Overview of Included Studies

The literature search was conducted in the Scopus and PubMed databases, covering publications from 2017 to 2024. A total of 2715 records were identified (1843 from Scopus and 872 from PubMed). After removing 372 duplicates and 19 reviews, 2324 records remained for title and abstract screening. Of these, 1743 records were excluded for not meeting the initial criteria.

**Figure 1.**
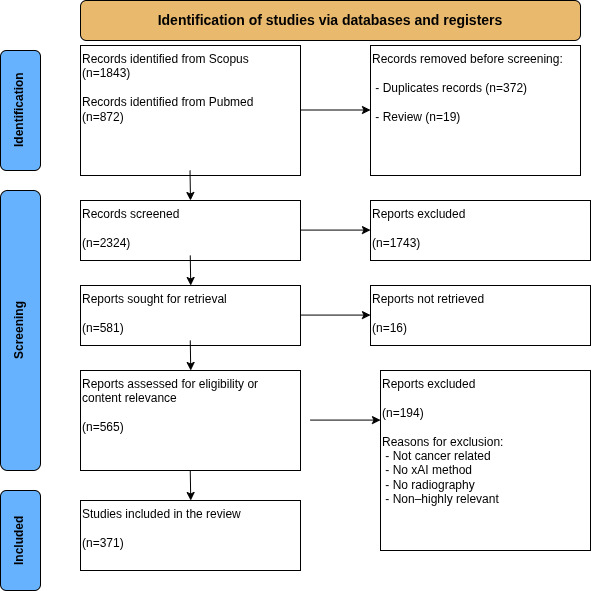
PRISMA (Preferred Reporting Items for Systematic Reviews and Meta-Analyses) 2020 flow diagram illustrating the selection process for the studies investigated. The search identified peer-reviewed studies published between 2017 and 2024 in Scopus and PubMed. After duplicate removal and exclusion of reviews, 2324 records were screened and 371 studies met the inclusion criteria. xAI: explainable artificial intelligence.

Subsequently, 581 reports were sought for retrieval, of which 16 could not be retrieved. A total of 565 reports were assessed for eligibility. Upon full-text review, 194 studies were excluded as they did not meet the specific inclusion criteria, primarily due to a lack of focus on cancer diagnosis, the absence of demonstrable xAI methods, or the use of nonradiological data. Finally, 371 studies were included in the review (Figure 1).

### Summary of Study Characteristics

To provide a comprehensive overview of the included literature, Tables 1 and 2 summarize the key characteristics and methodological approaches of all 371 studies. These tables present the collected data on temporal distribution, cancer types, imaging modalities, dataset accessibility, xAI implementation approaches, and validation practices, offering a systematic mapping of the current landscape of xAI in cancer imaging research.

**Table 1.** Study characteristics of included studies (N=371). Temporal distribution, cancer types, imaging modalities, and dataset accessibility among the 371 included studies. Studies spanned 2017‐2024, with most published in 2024 (n=200, 53.9%).

Characteristic and category	Studies (N=371), n (%)
Publication period	
2017‐2023	171 (46.1)
2024	200 (53.9)
Cancer type	
Breast	112 (30.2)
Lung	87 (23.5)
Brain or CNS^a^	56 (15.1)
Prostate	22 (5.9)
Thyroid	19 (5.1)
Head and Neck	16 (4.3)
Liver (Hepatocellular)	14 (3.8)
Pancreatic	7 (1.9)
Colorectal	5 (1.3)
Renal or kidney	5 (1.3)
Cervical	4 (1.1)
Other^b^	24 (6.5)
Imaging modality	
CT^c^	139 (37.5)
MRI^d^	104 (28)
Ultrasound	63 (17)
Mammography	46 (12.4)
X-ray	8 (2.2)
PET^e^-CT	8 (2.2)
Other or multiple^f^	3 (0.8)
Dataset accessibility	
Open	149 (40.2)
Mixed or combined	100 (26.9)
Not open	85 (22.9)
Uncertain	29 (7.8)
Synthetic or custom	8 (2.2)

a CNS: central nervous system.

b Includes rare malignancies and multidisease studies (each <1%): bone, stomach, ovary, esophagus, hematologic, skin, endometrial, bladder.

c CT: computed tomography.

d MRI: magnetic resonance imaging.

e PET: positron emission tomography.

f Some studies used multiple imaging modalities.

**Table 2.** xAI^a^ methods, validation, and reproducibility indicators (N=371). Distribution of xAI approaches, modeling methods, validation practices, code availability, and DSS^b^ integration.

Characteristic and category	N=371, n (%^c^)
xAI approach type	
Post hoc	305 (82.2)
Hybrid	45 (12.1)
Intrinsically interpretable	21 (5.7)
Post hoc method subcategories^d^	
Visualization-based	163 (53.4)
Feature relevance	111 (36.4)
Text explanations	7 (2.3)
Explanation by example	6 (2)
Simplification or surrogate	5 (1.6)
Combination (multimodal)	13 (4.3)
Primary modeling approach	
Deep learning	260 (70.1)
Classical ML^e^	67 (18.1)
Hybrid	37 (10)
Emerging or other	7 (1.9)
xAI validation status	
Validation reported^f^	193 (52)^g^
Expert or user-based	104 (28)^g^
Mixed methods	74 (38.3)^g^
Quantitative metrics	10 (5.2)^g^
Domain or clinical knowledge	8 (4.1)^g^
No validation reported	178 (48)
Code availability	
Code shared	65 (17.5)
Code not shared	280 (75.5)
Not specified	26 (7)
DSS integration	
DSS integration reported	45 (12)
No DSS integration	321 (86.5)
Not specified	5 (1.3)

a xAI: explainable artificial intelligence.

b DSS: decision support system.

c Percentages calculated as proportion of 305 post-hoc studies.

d Post hoc subcategories are mutually exclusive; “Combination” indicates use of ≥2 post hoc methods

e ML: machine learning.

f For validation subcategories, percentages in parentheses represent the proportion of the 193 validated studies; percentages can exceed 100% as categories are not mutually exclusive.

g Of validated.

Table 1 presents the distribution of studies by publication period, cancer type, imaging modality, and dataset accessibility. Most studies were published in 2024 (n=200, 53.9%). Breast cancer was the most frequently studied malignancy (n=112, 30.2%), followed by lung cancer (n=87, 23.5%), and brain or central nervous system (CNS) tumors (n=56, 15.1%). CT (n=139, 37.5%) and MRI (n=104, 28%) were the most commonly used primary imaging modalities. Regarding data accessibility, 149 studies (40.2%) used open datasets, while 85 (22.9%) relied on not-open institutional or private sources.

Table 2 summarizes xAI implementation approaches and validation practices. Post hoc explainability methods dominated (n=305, 82.2%), followed by hybrid approaches (n=45, 12.1%), and intrinsically interpretable models (n=21, 5.7%). Approximately half of the studies reported some form of xAI validation (n=193, 52%), most commonly expert or user-based evaluation (n=104, 28% of all studies); validation categories were not mutually exclusive, and totals may exceed 193. Code sharing remained limited (n=65, 17.5%), and integration into clinical DSS was reported in 45 studies (12.1%).

### Study Characteristics

#### Cancer Types

xAI methods have been applied to a wide range of cancer types, demonstrating their versatility in oncologic imaging. Breast cancer was the most extensively studied (n=112/371, 30.2%), followed by lung cancer (n=87/371, 23.5%) and brain or CNS tumors (n=56/371, 15.1%). Together, these three categories accounted for nearly 70% (255/371) of all reviewed studies, consistent with their global incidence and mortality burden and with previous bibliometric analyses of AI in oncology [1,43].

Moderately represented cancer types included prostate (22/371, 5.9%), thyroid (19/371, 5.1%), head and neck (16/371, 4.3%), and liver (hepatocellular and related; 14/371, 3.8%). Smaller groups addressed pancreatic (7/371, 1.9%), colorectal (5/371, 1.3%), renal or kidney (5/371, 1.3%), and cervical (4/371, 1.1%) malignancies. Other sites, such as stomach, bone, ovary, esophagus, hematologic, skin, endometrial, and bladder cancers, were examined in 5 or fewer studies (<1% each).

Within the “*Other”* category (n=24), 6 studies (6/371, 1.6%) addressed multidisease or nonorgan-specific contexts, including salivary gland tumors, metastasis or lymph node prediction, gastrointestinal stromal tumors, and mixed cancer cohorts that combined, for example, breast, brain, and cardiovascular imaging. Collectively, these studies highlight ongoing efforts to develop generalizable and cross-site explainable AI frameworks that extend beyond single-organ applications.

#### Imaging Modalities

**Figure 2.**
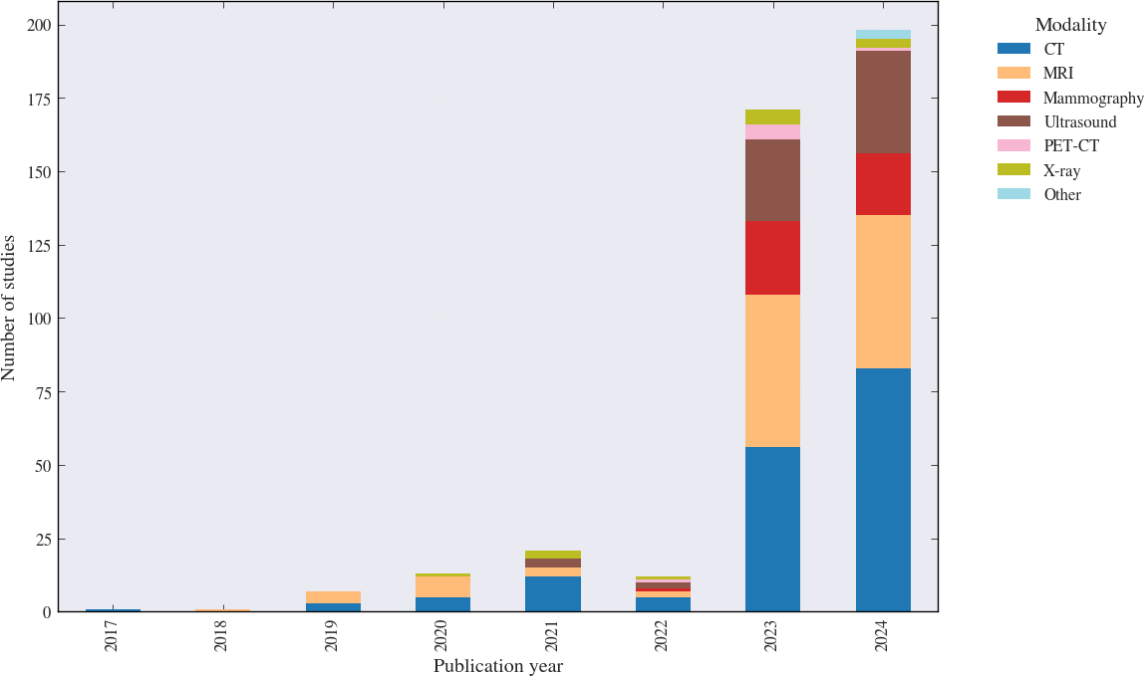
Distribution of imaging modalities used in explainable artificial intelligence studies for cancer imaging (2017‐2024; N=371). Stacked bars indicate the number of studies per publication year by modality. CT: computed tomography; MRI: magnetic resonance imaging; PET: positron emission tomography.

Across all included studies, CT (n=139, 37.5%) and MRI (n=104, 28%) were the most common primary modalities, followed by ultrasound (n=63, 17%) and mammography (n=46, 12.4%). Multimodal designs were present but were assigned to a primary modality (or “Other or multiple,” n=3, 0.8%) for counting in Table 1. X-ray and PET-CT were less common (n=8 each, 2.2%) but remain relevant for specific clinical contexts, particularly thoracic and metastatic imaging (Figure 2).

Over time, CT remained the most common modality, particularly in 2023 (56/171, 32.7%) and 2024 (83/200, 41.5%; Figure 2). MRI remained the second most common modality across the period. Ultrasound and mammography contributed a substantial minority of studies, particularly in later years, reflecting continued interest in breast-focused imaging and multimodality-based workflows. PET-CT and x-ray appeared less frequently but remained present in clinically relevant contexts.

### Dataset Availability and Composition

The datasets used in the 371 reviewed studies came from a wide range of sources, including clinical repositories, public challenges, and institutional imaging archives (Figure S1 in Multimedia Appendix 4; Table 3). This diversity demonstrates the broad scope of AI applications in oncology and reflects varying levels of accessibility and reproducibility across studies. Dataset availability was categorized into 5 groups: open (publicly accessible repositories), not open (institutional or private sources), mixed or combined (both open and private sources), synthetic or custom (purpose-built datasets), and uncertain (insufficient information).

**Table 3.** Accessibility of datasets used in studies applying artificial intelligence to cancer imaging (2017-2024; N=371). The corpus includes major cancers (breast, lung, brain, liver, and prostate) from multiple continents.

Dataset accessibility category	Studies, n (%)
Open	149 (40.2)
Mixed or combined	100 (26.9)
Not open	85 (22.9)
Uncertain	29 (7.8)
Synthetic or custom	8 (2.2)
Total	371 (100)

Most studies relied on open or mixed data sources. Open datasets accounted for 149 of 371 (40.2%) studies, making up the largest single group. These included established repositories such as LIDC-LDRI, the Cancer Imaging Archive (TCIA), the Cancer Genome Atlas (TCGA), CBISDDSM, and public competition datasets like PROSTATEx and AAPM 2017, all directly cited in the reviewed works. Openly available datasets are essential for ensuring reproducibility, benchmarking, and collaborative validation across institutions.

In contrast, not-open datasets comprised 85 of 371 (22.9%) studies, typically based on patient data from hospitals and medical centers (eg, Harbin Medical University, Ruijin Hospital, and Asan Medical Center). These datasets are inaccessible to external researchers, limiting reproducibility despite their clinical authenticity.

A substantial proportion, 100 of 371 (26.9%) studies, used a mixed or combined approach, integrating public datasets with proprietary clinical data to improve external validity and increase sample diversity. For example, some studies combined TCGA or TCIA datasets with internal cohorts for independent testing.

A smaller subset, 8 of 371 (2.2%) studies, relied on synthetic or custom datasets created specifically for research purposes, such as simulated imaging phantoms or institution-specific multimodal collections (eg, Credence Cartridge Radiomics phantom). These datasets often integrate imaging, clinical, and demographic data tailored to specific research questions.

Finally, 29 of 371 (7.8%) studies provided unclear or incomplete information about their data sources, highlighting ongoing challenges in dataset transparency and reporting standards.

### Modeling Approaches

#### Methodological Note

Because some studies used multiple models, we assigned a single main modeling approach to each study. Categories are mutually exclusive: ML, DL, hybrid, or “atypical.” We labeled a study as hybrid when prediction relied on both DL and classical ML (eg, a Convolutional Neural Network [CNN] feature extractor with an extreme gradient boosting [XGBoost] for inference) or when the architecture explicitly integrated multiple families; otherwise, we labeled by the dominant predictive component (eg, end-to-end CNN=DL, radiomics plus gradient boosting=classical ML). Counts, therefore, sum to n=371 and refer to studies, not model instances.

#### Overview

In the majority of studied articles (n=260/371, 70.1%), DL architectures are used (Figure S2 in Multimedia Appendix 4 and Table 4). In most cases, they serve as the main analytical components of the analyzed studies; in other cases, they are part of hybrid pipelines where classical ML algorithms are used for secondary classification or feature aggregation. Within DL, CNN-based families (eg, Residual Network, Visual Geometry Group, and Densely Connected Convolutional Network) remain dominant for image-level tasks, U-Net variants are the default for segmentation, and transformer or attention architectures rose notably in 2023, aligning with self-attention maps as native interpretability; CNNs still constitute the largest DL subgroup (149/260, 57.3%). In hybrid CNN-to-ML setups, the DL block extracts spatial or contextual features while the ML block (eg, support vector machine [SVM], random forest [RF], and XGBoost) provides interpretable decision rules or feature rankings.

**Table 4.** Distribution of primary modeling approaches among examined studies (2017-2024; N=371).

Primary modeling approach	Studies, n (%)
Deep learning	260 (70.1)
Classical machine learning	67 (18.1)
Hybrid approaches	37 (10)
Emerging or other	7 (1.9)
Total	371 (100)

Classical ML algorithms (67/371, 18.1%) are still used frequently, mostly in analyses focused on radiomics or as comparative baselines. Models commonly selected for interpretability include RFs and SVMs (feature importance and margin or kernel effects), while since 2023 gradient-boosting families (Categorical Boosting, Light Gradient Boosting Machine, and XGBoost) are frequently used in ensemble or multimodal fusion pipelines because they connect directly to structured radiomics or clinical variables and expose feature attributions.

The number of hybrid approaches (37/371, 10%), including CNN-ML ensembles, multitask frameworks, and Concept Bottleneck Model architectures has increased between 2023 and 2024. Hybrid approaches integrate complementary model families or data sources to balance performance with interpretability. Typical patterns include CNN and ML pipelines, where a deep encoder produces compact features that a transparent learner (eg, SVM, RF, and gradient boosting) turns into decision rules or feature rankings. Overall, these designs emphasize embedding interpretability within the training pipeline rather than relying solely on post hoc explanations.

A small portion of studies (7/371, 1.9%) use modeling approaches that are less common in current practice. Notably, concept and prototype-based designs (eg, Concept Bottleneck Models, ProtoPNet, and Breast Imaging Reporting and Data System [BI-RADS]-NET) and architectures with explicit causal mechanisms (eg, Causeg-Net and BDStable-Net) aim to anchor model learning to clinically meaningful factors and human-understandable concepts, providing built-in transparency.

### Explainability Methods in Cancer Imaging

#### Terminology Landscape in the Reviewed Studies

A qualitative analysis of the corpus identified 6 recurring categories in terminology (Figure S3 in Multimedia Appendix 4). Most usage is concentrated in the categories of interpretable or interpretability and explainable or explainability. Trustworthy and transparent also appear, but as secondary themes. Other labels, such as reliable or visualization, form a “long tail.” Because the key terms for retrieval were both explainable and interpretable, these observations are descriptive rather than unbiased frequency counts. The takeaway is that xAI vocabulary is diverse and often used interchangeably in literature. Therefore, we report this mapping to support replication and facilitate future searches.

#### Distribution of Explainability Types

Analysis across 371 studies published from 2017 to 2024 showed that post hoc xAI methods were dominant, used in 82.2% (n=305) of all studies (Figure S4 in Multimedia Appendix 4 and Table 5). Most of these studies used post hoc as the primary means to add transparency to their predictive models. Studies using inherently transparent models, such as those with built-in structures (eg, DTs and linear coefficients) that enable transparency by design, accounted only for 5.7% (n=21) of studies. Hybrid methods accounted for 12.1% (n=45) of studies, combining inherently interpretable models with post hoc techniques to provide more robust or complementary explanations.

**Table 5.** Distribution of explainable artificial intelligence methods across included studies.

Category	Studies, n (%)
Post hoc	305 (82.2)
Visualization-based	163 (53.4)
Feature relevance	111 (36.4)
Combination	13 (4.3)
Text explanation	7 (2.3)
Explanation by example	6 (2)
Explanation by simplification	5 (1.6)
Hybrid	45 (12.1)
Ante-hoc	21 (5.7)
Total	371 (100)

Post hoc approaches dominated (n=305, 82.2%), followed by hybrid (n=45, 12.1%) and ante hoc (intrinsic) methods (n=21, 5.7%). Within the post hoc group, visualization-based explanations, such as saliency maps and activation heatmaps, were the most prevalent (n=163, 53.4%), followed by feature relevance approaches including SHAP and layer-wise relevance propagation (n=111, 36.4%). Combination (multimodal) post hoc strategies accounted for 4.3% (n=13). Other categories, such as text explanations (n=7, 2.3%), explanations by example (n=6, 2%), and explanations by simplification (n=5, 1.6%), were comparatively infrequent, underscoring the dominance of visual and feature-based interpretability techniques in current xAI research.

The temporal evolution of explainability methods from 2017 to 2024 reveals a clear and notable trend. Post hoc methods have consistently remained the dominant approach; however, the composition of the methods used has changed. Whereas visualization-based methods, such as Grad-CAM, were previously most common, they are now increasingly rivaled by more recently available feature-perturbation methods, such as SHAP, which have even become dominant in some years.

The extent to which the study authors clearly defined the goals for explainability varied throughout the study period. Early studies were more likely to define explainability goals broadly (eg, “to improve interpretability” and “to provide explanations”) without specifying the particular clinical applications or potential user groups. In contrast, more recent studies increasingly define specific objectives (eg, to foster clinician confidence, to make model output consistent with diagnostic standards, such as BI-RADS, and to enable visual validation of lesion location).

#### Inherently Interpretable or Transparent Models

In only 21 of 371 studies (approximately 5.7%), researchers used models designed to be interpretable from the beginning, rather than explaining them retrospectively, indicating that fully intrinsic interpretability remains rare.

The largest group of studies (9/21) achieved interpretability by imposing structural or architectural constraints within deep models. Examples include the hierarchical semantic CNN or hierarchical semantic network for lung nodule malignancy [44], Thyroid Imaging Reporting and Data System–based multitask networks for thyroid nodules [45], prototype-based CNNs for mammography [46], and lesion-level risk aggregation models for metastatic lung cancer prognosis [47]. In these cases, the model provides clinically relevant information, such as nodule shape or margin [48], assigns specific per-lesion risk scores [47], or presents prototypical patterns as part of its standard inference process [46].

In a second subset (3/21), studies used generalized additive or sparse linear models, such as Explainable Boosting Machines and least absolute shrinkage and selection operator–regularized linear or logistic regression, for prognosis of treatment response (local failure in head and neck cancer [49]; 3-year overall survival in early-stage non–small cell lung cancer [50]) and prediction of tumor markers in kidney cancer [51]. Feature-wise contribution functions and sparse coefficients on clinically meaningful features serve as the built-in explanations of these models.

In total, 5 studies (5/21) used transparent, feature-engineered pipelines, for example, for prostate cancer segmentation [52], or liver cancer treatment monitoring [53], and tumor classification in breast MRI [54], focusing on explicit feature rankings. Furthermore, 2 studies defined their interpretability in terms of biologically grounded phenotypes, such as radiomic factors linked to CD8+ T lymphocyte infiltration and tumor microenvironment phenotypes, using these as model inputs instead of opaque latent features [55,56].

Moreover, 2 other studies (2/21) were based on probabilistic and fuzzy models (fuzzy classification for radiotherapy toxicity and Bayesian multitask learning regression), providing global interpretability through human-readable fuzzy rules or task-specific linear mappings between predictors and clinical targets. Rule-based and symbolic learners were also present in this xAI category (2/21; rule-fit for chemoradiation PET response in non–small cell lung cancer [57] and grammatical evolution classifiers for breast cancer [58]). They generated explicit human-readable rules and formulas.

Studies in this category generally adopted transparent models to meet clinical interpretability requirements, such as providing explicit feature importance rankings from radiomics features or feature-outcome correlation plots, rather than relying on visual explanations.

#### Post Hoc Explainability Approaches

##### Overview

Within the post hoc subset (n=305), visualization-based explanations were the most common (163/305, 53.4%), followed by feature relevance methods, such as SHAP and LIME (111/305, 36.4%). Text-based explanations (7/305, 2.3%), explanation-by-example approaches (6/305, 2%), and simplification or surrogate-based methods (5/305, 1.6%) were comparatively rare. A subset of studies used combination (multimodal) post hoc strategies (13/305, 4.3%), integrating 2 or more post hoc explanation types to provide complementary perspectives on model behavior.

##### Visualization-Based Methods

The most common class includes visualization techniques (163/305, 53.4%). Representative methods include Grad-CAM, saliency maps, and attention heat maps. These techniques are well-suited to CNN-based architectures, such as Res-Net, Dense-Net, U-Net, and their attention-enhanced variants, and are commonly used in radiology due to their intuitive and spatially localized results [59,60].

The main motivation for these methods is to highlight image regions that influence the networks’ decisions and align them with the radiologist’s reasoning, thus promoting clinical confidence. They are especially common in CNN-based classification and segmentation studies, where visual inspection provides intuitive correspondence with tumor or lesion regions. For example, studies have used Grad-CAM to highlight tumor subregions in breast [61-63], brain [64-66], prostate [67], and thyroid imaging [68,69].

Grad-CAM or CAM approaches represent the majority (73/163, 44.8%), followed by saliency or heatmap techniques, and other visualization-based strategies. An important limitation reported in the investigated studies [70] is the subjective nature of heatmap interpretation, which underscores the need for standardized metrics. Nevertheless, the emergence of variants, such as Full-Grad or attention-based overlays, reflects the ongoing refinement of visualization-based xAI approaches [71].

##### Feature-Relevance Methods

The second large category includes feature attribution techniques (111/305, 36.4%), in particular SHAP. For reporting, we merged in this category the studies that fall under the category of *local explanations* (eg, LIME; n=3). Although Arrieta et al [34] define them as distinct subcategories*,* in practice, there is overlap in their techniques and objectives. Therefore, for clarity and alignment with the methods used in the reported results, we combined them into a single *feature relevance* category.

These approaches assign importance scores to individual input features, such as radiomic markers [72,73], clinical attributes, or embedded feature vectors [74,75], and are particularly useful when DL models are applied to multimodal or structured data [76-78], including combinations of imaging and clinical information [79]. Most studies use CNN-based and feature-enhanced architectures (eg, Res-Net, VGG16, and U-Net), often in combination with attention modules or radiomic pipelines for structured inputs.

##### Simplification or Surrogate Models

A small group of studies (5/305, 1.6%) use simplification techniques that aim to improve interpretability by creating surrogate or human-understandable models that approximate the behavior of complex DL systems. These methods (eg, rule extraction, fuzzy inference systems, and contour-based symbolic mappings) translate model logic into structured formats that clinicians can interpret more easily. The underlying architectures are usually CNN-based classifiers. Although methodologically sound, their adoption remains limited, mostly conceptual or in proof-of-concept stages due to concerns over surrogate fidelity and reproducibility [80].

Examples include a study by Contreras et al [81], where the authors derive rule sets to mimic a black box model and explain human papillomavirus diagnosis. In [82], the model integrated a Takagi-Sugeno-Kang fuzzy system to convert feature activations into interpretable fuzzy rules. In another study [83], mathematical curve modeling was used to approximate region of interest boundaries in a clinically meaningful way. Studies reported that these simplification methods can improve interpretability while achieving comparable or higher predictive performance than the black box alternatives [84,85].

##### Text Explanations

Text explanation category methods were used in only a few studies (n=7), typically through post hoc textual outputs designed to complement predictions with human-interpretable reasoning. In several cases, explanations were based on clinical descriptors. For instance, semantic outputs aligned with BI-RADS criteria were used in breast cancer classification models to enhance interpretability for clinicians [86,87]. A graphical user interface–driven explanation system providing diagnostic and treatment suggestions was implemented for airway disease classification using CNN models [88].

With the advent of large language models, studies that integrate them in xAI pipelines have emerged. In one, GPT-4 (OpenAI) was evaluated for thyroid ultrasound analysis, which decomposed the reasoning behind each diagnostic output [89]. Furthermore, 2 studies linked imaging features to biological information—one associated radiomics with long noncoding RNA expression [90], while another correlated CT-derived features with biological pathways using gene set variation analysis [91].

##### Explanation by Example

In total, 6 studies used example-based explanations through prototype reasoning or counterfactual generation. In the first category, we have studies that used prototype-based explainers, such as the study by Yang et al [92], which justify the model’s prediction by showing image regions that match learned “prototypes” of lesions (ie, the nearest positive examples aligned with clinical cues). The other category, using counterfactuals [93], includes studies that answer the question “what minimal change would change the model’s outcome?,” thus generating contrastive examples in order to reduce spurious cues and focus attention on the lesion. Another example in the counterfactuals category [94] altered slightly imaging, clinical, and molecular variables to identify drivers for treatment response. A common thing in this category is that all methods were model-specific and local, that is, the predictions were generated per instance.

##### Multimodal Explanation Strategies

A total of 13 studies used a combination of post hoc xAI methods from different subcategories, most commonly feature relevance and visualization. Feature relevance methods, such as SHAP and LIME, were used to assign importance scores to input features, while visualization techniques, like Grad-CAM and attention maps, highlighted spatial or contextual cues within medical images. Typically, visualization-based methods, such as Grad-CAM, are model-specific; however, SHAP and LIME are model-agnostic, allowing for flexible integration of these techniques across various architectures.

The use of both types of techniques was common, and they were often applied together to provide global and local insights. For example, SHAP and Grad-CAM were used in multimodal or ensemble frameworks to interpret diagnostic and prognostic models [95-98]. On the other hand, attention maps and risk heatmaps were used alongside feature attribution techniques in transformer and graph-based networks [99,100]. Overall, these studies indicate a growing trend towards hybrid xAI approaches that use multiple perspectives to promote transparency.

### Explainability Practices by Imaging Modality

CT and MRI were the most common modalities, with 139 (37.5%) and 104 (28.0%) studies, respectively, followed by ultrasound (n=63, 17%) and mammography (n=46, 12.4%). Validation practices varied by modality. Studies using ultrasound and mammography reported higher rates of xAI component validation (72.2% and 76.9%, respectively) compared with CT and MRI (44.8% and 43.1%, respectively). Expert-based validation showed a similar trend—ultrasound (61.1%) and mammography (61.5%) exceeded CT (32.1%) and MRI (25.5%).

Preferences for explainability methods also differed by modality. Most mammography studies used Grad-CAM (19/46, 42.3%), while SHAP was used in approximately 7% (3/46). In contrast, CT studies used SHAP more often (50/139, ~35.8%) than Grad-CAM (29/139, 20.9%). MRI studies showed a more balanced use, with Grad-CAM (30/104, ~28.4%) and SHAP (22/104, ~22.5%).

### Validation of Explainability

#### Overview

In this review, we examined how studies validated their explainability components, the metrics and strategies adopted, and the extent of user involvement. Among all included studies, 193 (52%) reported at least 1 form of xAI validation. Because some studies used multiple validation strategies, categories overlap, and totals can exceed 193. *Expert or user-based validation* was reported in 104 studies (28% of all studies; 53.9% of validated studies). *Mixed methods validation* was reported in 74 studies (19.9% of all studies; 38.3% of validated studies). *Quantitative metrics* were reported in 10 studies (2.7% of all studies; 5.2% of validated studies), and *domain or clinical-knowledge validation* in 8 studies (2.2% of all studies; 4.1% of validated studies).

#### Validation From Experts and Users

The majority of studies (n=104/193, 53%) based their validation primarily on expert feedback obtained from doctors, radiologists, or clinicians. They assessed whether the explanations aligned with medical experts’ reasoning during diagnosis. This was achieved through interviews with them, surveys of readers, or usability tests to determine if the AI explanations were logical and understandable. Even when researchers did not explicitly label their studies as “validation of xAI,” many included some form of evaluation of the usability and reasoning of the AI’s explanations, contributing to the overall objective of validating the AI’s explanatory ability.

#### Quantitative Measurement and Model Comparison

Fewer studies (10/193, approximately 5%) relied on indirect assessment of explanation quality using performance metrics or model comparisons. In particular, studies reported using various metrics to measure the quality of AI-generated explanations. Among the most common metrics were area under the curve (AUC), precision-recall AUC, and feature ranking consistencies. However, most of the time, these metrics assess the quality of the model’s predictions rather than the explanations themselves.

#### Validation Related to Specific Domains or Clinical Applications

Some studies validated explanations by checking their consistency with current clinical and/or biological knowledge (8/193, 4%). For example, they verified agreement between the model’s predictions and histopathological characteristics; compared the model’s explanatory features with radiologists’ accepted standards to ensure consistency; or tested whether the explanatory features remained consistent across all data partitions. All these methods help demonstrate the validity of the clinical application of the xAI system in medicine.

#### Mixed Methods Validation

In addition to the above types of validation, some studies (74/193, 37.7%) combined 2 or more methods to validate the quality of AI explanations. Most often, this mixed methods approach involved combining expert feedback with one of the 3 types of quantitative validation described previously. By doing so, the authors aimed to balance the need for objectivity in explanation quality with the need to understand experts’ perspectives on trustworthiness and usability.

For example, studies such as the one by Nowakowska et al [101] used SHAP-based feature importance analysis alongside expert radiologist review to validate the clinical coherence of model explanations. Finally, another study showed that AUC-based model performance can be triangulated with reader-study feedback to validate the alignment of visual explanations with diagnostic reasoning [48,102].

### Fairness and Bias

Beyond validation, trustworthy explainable AI in health care also depends on fairness and bias mitigation. While explainability improves interpretability, it does not inherently guarantee equitable or unbiased outcomes. Only a limited number of studies explicitly assessed bias within their explainability frameworks or examined how explanations might vary across patient subgroups [51,102,103]. The intersection of explainability and fairness remains underexplored.

### Code Availability and Decision-Support Integration

#### Code Availability

**Figure 3.**
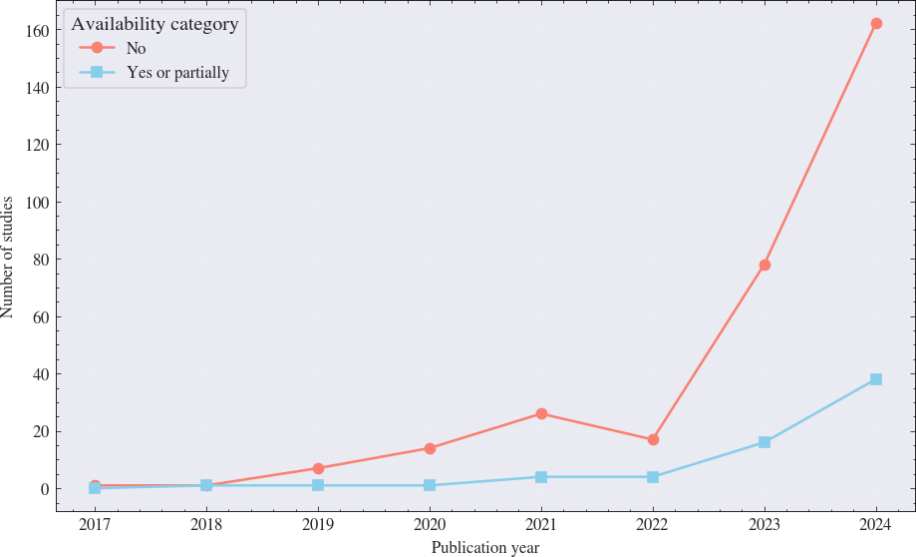
Time trend of code availability among included explainable artificial intelligence cancer imaging studies (2017‐2024; N=371).

Based on the review of 371 studies, most of them did not share their code (Figure 3). Of the 371 reviewed studies, only 17.5% (65/371) reported sharing their full or partial implementation via a platform (such as GitHub) or upon request. In contrast, 82.5% (306/371) provided no access to their source code, a figure that includes studies where no code availability information was reported, which were confirmed through subset sampling to represent a lack of shared implementation. The failure to make source code available is a major hindrance to reproducing any findings, preventing peers from verifying the work and limiting advancements through collaboration in the field.

Despite the overall low transparency, a longitudinal analysis reveals a positive shift in open science practices. From 2017 to 2023, only 15.8% (27/171) of studies provided public or partial access to their code. However, among studies published in 2024, this proportion rose to 19% (38/200). While the volume of “no” results is increasing in absolute terms due to the explosion of xAI research, the relative rate of code sharing is showing a steady upward trend.

#### DSS Integration

**Figure 4.**
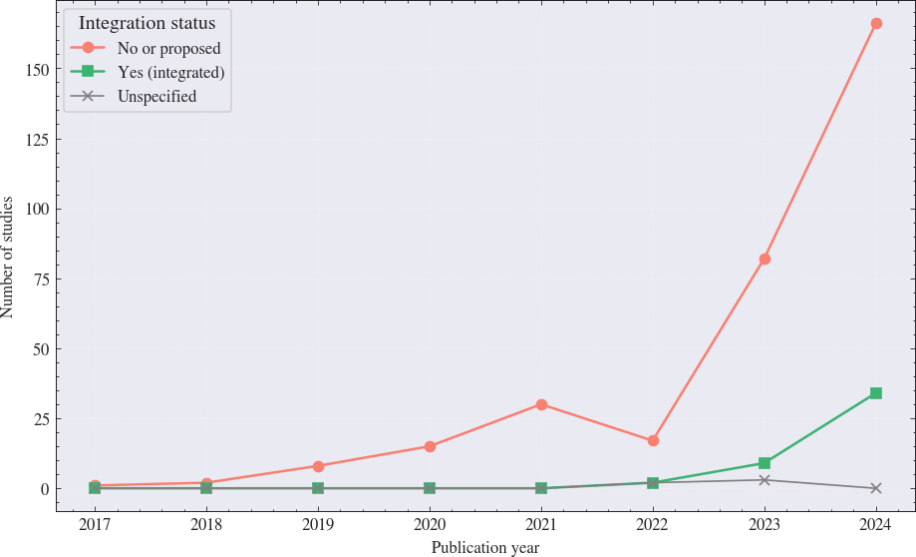
Integration of Explainable artificial intelligence models into decision support systems among included studies (2017‐2024; N=371).

Out of 371 studies, only 45 (12.1%) reported full or partial DSS integration, while 321 (86.5%) did not, and 5 (1.3%) were unspecified (Figure 4). A temporal comparison shows a modest increase in reported integration; only 11 out of 171 studies (6.4%) from 2017 to 2023 mentioned DSS use, compared with 34 out of 200 (17%) in 2024. Among all the 2024 studies, the majority discussed potential or planned integration (69/200, 34.5%), often in the form of prototypes, user interfaces, or frameworks intended for future translation, while fewer describe actual deployment within clinical workflows (34/200, 17%).

### Summary of Evidence Patterns

Across the 371 included studies, research activity was concentrated in breast, lung, and brain or CNS cancers (together 255/371, approximately 70% of studies). DL was the dominant primary modeling approach (260/371, 70.1%), and post hoc explainability methods were used in most studies (305/371, 82.2%), whereas ante hoc (intrinsic) explainability approaches were uncommon (21/371, 5.7%). Explainability validation was reported in 52% (193/371) of studies, most frequently expert or user-based evaluation. Reproducibility and translation indicators were limited; 17.5% (65/371) of studies reported code availability, and 12% (45/371) reported DSS integration; an additional 18.6% (69/371) proposed clinical integration without reporting deployment.

## Discussion

### Summary of Findings

In this review, we examined how explainability is integrated in cancer imaging AI studies, how explanation techniques are assessed and reported, and the extent to which current reporting facilitates reproducibility and clinical use. Overall, explainability in AI studies is primarily implemented through post hoc methods, while intrinsically interpretable and hybrid approaches remain relatively uncommon. Thus, in much of the literature, transparency is treated as an add-on to predictive modeling rather than as a fundamental aspect of model design.

Furthermore, we identified significant variability in the appraisal of xAI methods. Many studies used qualitative or plausibility-based assessments, and expert or user feedback was the most reported method for evaluating explainability. However, these evaluations were generally conducted without more rigorous methods for testing the fidelity and consistency of explanations against existing domain knowledge. Conversely, the use of formal metrics for measuring explanation quality varied significantly between studies.

Our results show limited support for indicators for reproducibility and translation to clinical settings. Code sharing was infrequent, data access was often restricted, and only a small number of studies demonstrated integration of their models into clinical decision-making systems. These findings suggest that although there is an expanding body of evidence related to xAI, further development is needed to standardize the evaluation of explainability and to establish stronger clinical relevance.

### Interpretation and Comparison With Previous Literature

#### Explainability Trends and Methodological Implications

Consistent with previous reviews in medical imaging, the dominance of post hoc explainability methods, including Grad-CAM and SHAP, reflects a trend similar to that observed in earlier literature [104,105]. This dominance is largely due to the relative ease of implementation and versatility of visual attribution methods, as well as their compatibility with a wide range of model architectures, particularly CNNs [106,107]. However, these methods generally provide qualitative explanations, which can contain a subjective side and be significantly affected by perturbation [104,108].

Although the majority of studies reporting xAI validation used some form of expert feedback, these assessments were typically limited to plausibility judgments, asking whether explanations appeared reasonable, rather than a systematic evaluation of whether model attributions corresponded to established clinical or biological knowledge.

The widespread use of SHAP across studies, driven by its ease of access and model-agnostic applicability, should be interpreted cautiously when evaluating clinical confidence. Fewer than one-third of the studies that employed SHAP included expert-based validation to assess alignment with clinical reasoning. This is notable given evidence that SHAP-derived attributions may have limited fidelity to actual model behavior [104], suggesting that technical interpretability should not be automatically equated with clinical transparency or alignment with radiological cognition.

As noted in our results, there appears to be limited adoption of inherently interpretable models, such as Explainable Boosting Machine or generalized additive model, which may be attributed to the perception—and empirical documentation—of an accuracy-interpretability tradeoff, with interpretable models showing a consistent 5%‐7% AUC penalty compared with black box approaches [104]. At the same time, the emergence of hybrid models that combine DL with traditional ML classifiers suggests growing interest in architectures that offer improved transparency while maintaining high model performance. This progression reflects trends observed in other recent surveys of explainable medical AI [109].

The stated purposes of explainability in the reviewed studies became more specific over time, shifting from general descriptions to articulating concrete clinical objectives. This trend may reflect the field’s maturation from demonstrating technical feasibility to addressing how explanations should be designed for clinical utility.

#### Modality-Specific Considerations

We identified CT and MRI as the two most widely used modalities in our study, although the rate at which xAI methods are validated and their application varies greatly depending on the modality. In the modality-specific subset analysis, we found that ultrasound and mammography studies reported validation of xAI more frequently than CT and MRI studies. There were also differences in method preference among the modalities. For example, mammography-based studies used Grad-CAM more often, while CT-based studies used SHAP more frequently. These differences in modality-specific application suggest that xAI may not be universally applied. Instead, xAI design and validation procedures will likely need to be tailored to each specific modality and workflow [110,111].

#### Validation Practices and Their Limitations

While approximately half of the reviewed studies reported some form of explainability validation, the approaches and rigor varied considerably. Expert-based validation predominated, typically involving radiologists or clinicians assessing whether explanations appeared plausible or aligned with their diagnostic reasoning. However, very few studies implemented deeper feedback loops—whether from expert critique or from xAI evaluation outcomes—to iteratively refine model features or architectures. This pattern suggests that validation often serves as post hoc verification rather than as an integral component of model development.

Quantitative validation methods, although less common, were typically indirect—relying on performance metrics (AUC and accuracy) that assess prediction quality rather than explanation fidelity. Few studies used formal metrics specifically designed to evaluate explanation quality, consistency, or clinical utility. This methodological gap, combined with the lack of standardized frameworks for assessing explanations, leaves most validation efforts qualitative and subjective [110].

Several limitations emerged across the reviewed literature. Many studies acknowledged that their models lacked multicenter or external validation, limiting confidence in both predictions and explanations. Training on small or imbalanced datasets has been associated with instability in both model predictions [112,113] and attribution patterns [104]. Researchers also noted that heatmap interpretations remain subjective and may not correspond to clinical reasoning, risking misinterpretation by users lacking appropriate context [104]. Additionally, the complexity or opacity of some model architectures can render generated explanations less actionable in clinical settings.

Importantly, validation results were rarely used to modify model design. Explainability was typically incorporated after model development and validation were complete, a pattern consistent with the predominance of post hoc rather than in-model approaches observed across the broader medical AI literature [114,115]. This post hoc approach limits opportunities for iterative refinement and may contribute to overestimation of clinical readiness.

### Clinical Translation, Trustworthiness, and Reproducibility

Clinical translation of xAI remains limited due to multiple interconnected barriers. Although approximately half of the studies included some form of expert-based validation, this rarely extended beyond plausibility assessment to active clinical integration or workflow testing. This gap is consistent with previous criticisms that explanation methods do not necessarily reflect clinical reasoning and may lead to user misinterpretation [116]. Few studies included clinicians as active participants in validation, and even fewer incorporated clinical feedback to assess whether model explanations aligned with actual decision-making processes or improved workflow integration. Structured frameworks offer pathways for human-in-the-loop validation and user-centered evaluation, yet their adoption remains limited. The low percentage of reported DSS system deployments, along with the limited number of documented clinical applications, clearly shows that most systems are in the early stages of proof-of-concept models—disconnected from real-world clinical environments. However, the increase in studies conducted during the last year of this review that focus on clinical applicability indicates that the field is at a developmental stage where translation to clinical practice will be feasible.

Additionally, there are significant barriers to reproducibility. Code sharing was rare, and reliance on private or mixed datasets limits independent replication of findings. These patterns mirror challenges in the broader medical AI literature, where transparency, data governance, and external validation continue to be significant barriers to progress [117,118]. Similarly, fairness and subgroup analysis were rarely reported, leaving critical questions about algorithmic equity unanswered. In summary, while xAI methods are being widely adopted in research settings, limited validation rigor, minimal integration of clinical feedback, and insufficient attention to fairness may collectively contribute to an overestimation of clinical readiness relative to what current evidence supports.

### Limitations of This Review

There are 4 main limitations of this study. First, the studies we examined showed considerable variation in how explainability was defined and implemented, making the classification as either post hoc or intrinsically interpretable subjective, even though we used standardized definitions. Second, the search was limited to PubMed and Scopus and to studies published in English, which may have led to missing relevant studies, particularly those published in computer science journals. Third, because we examined only radiologic imaging, the generalizability of our findings to other biomedical imaging disciplines, such as histopathology or dermoscopy, is uncertain. Fourth, as the field of xAI is rapidly advancing, we did not include studies published after December 2024, and the observed growth in xAI-related publications may partly reflect changes in indexing practices or reporting standards rather than a true increase in research activity.

### Conclusions

This scoping review provides a comprehensive mapping of xAI methods across multiple cancer imaging modalities, addressing a gap left by previous reviews that focused on single modalities or isolated aspects of interpretability. By quantifying key indicators, including validation rates (52%), code availability (17.5%), and DSS integration (12.1%), this study offers an evidence base for researchers, clinicians, and regulators to understand the current state of xAI readiness for clinical use. The main opportunities to advance xAI in cancer imaging include validating current explainability methods—given that only 52% (193/371) of studies reported any form of explainability validation—improving data access, increasing the use of shared code, and fostering a culture of openness and trust. These elements of reproducibility and transparency are essential for developing reliable clinical AI models. Reporting guidelines, such as the Checklist for Artificial Intelligence in Medical Imaging [119], and broader frameworks (including Fairness, Universality, Traceability, Usability, Robustness, and Explainability [-AI] [118], and Developmental and Exploratory Clinical Investigations of Decision Support Systems [120]) structured requirements for transparency and trustworthy AI development; however, their full potential can only be realized through widespread adoption by the medical imaging community.

Progress in applying xAI to cancer imaging will also depend on the level of collaboration among developers, clinicians, and domain experts. Meaningful and clinically actionable explanations require clinically-informed design, early stakeholder involvement, and user-centered evaluation aligned with specific tasks and users (eg, radiologists, oncologists, or patients). Establishing common benchmark standards and standardized reporting will further support study reproducibility and comparison, and clinical translation. For regulators and institutions, these findings highlight that most xAI systems remain at the prototype stage. With standardized validation, collaborative development, and a multidisciplinary design, xAI systems can evolve from technically interpretable prototypes to clinically trustworthy tools that genuinely support decision-making in oncologic imaging.

## Supplementary material

10.2196/80645 Multimedia Appendix 1 Full search queries.

10.2196/80645 Multimedia Appendix 2 Data extraction schema.

10.2196/80645 Multimedia Appendix 3 Additional studies.

10.2196/80645 Multimedia Appendix 4 Additional figures.

10.2196/80645 Checklist 1 PRISMA-ScR checklist.

## Abbreviations

AI: artificial intelligence
AUC: area under the curve
BI-RADS: Breast Imaging Reporting and Data System
CNN: Convolutional Neural Network
CNS: central nervous system
CT: computed tomography
DL: deep learning
DSS: decision support system
DT: decision tree
Grad-CAM: gradient-weighted class activation mapping
LIME: local interpretable model-agnostic explanations
MeSH: Medical Subject Headings
ML: machine learning
MRI: magnetic resonance imaging
PET: positron emission tomography
PRISMA-S: Preferred Reporting Items for Systematic Reviews and Meta-Analyses Search Extension
PRISMA-ScR: Preferred Reporting Items for Systematic Reviews and Meta-Analyses extension for Scoping Reviews
RF: random forest
SHAP: Shapley Additive Explanations
SVM: support vector machine
TCGA: The Cancer Genome Atlas
TCIA: The Cancer Imaging Archive
xAI: explainable artificial intelligence
XGBoost: extreme gradient boosting
