# Supplementary material for: Explainable AI in Cancer Imaging: Scoping Review of Methods, Modalities, and Clinical Integration
Source: J Med Internet Res. 2026 May 20;28:e80645. doi: 10.2196/80645 (PMC13189567; doi:10.2196/80645)
Supplement: Multimedia Appendix 1 [file jmir-v28-e80645-s001.docx]

Multimedia Appendix 1 - Full Search Queries.docx

## This appendix provides the full search strategies used in the review, reported in accordance with the PRISMA-S guidelines. For each database, the platform, exact search execution date, and complete syntax are documented. Limits applied (e.g., language, publication type) reflect decisions described in the Methods section.

**PubMed query (NCBI)**

| **Platform/interface** | **NCBI** |
| --- | --- |
| **Search execution dates** | Initial search *:*  Updated search: |
| **Coverage intended** | 2017–2024 |
| Limits applied | English only; exclusion of reviews, systematic reviews, and meta-analyses |
|  | ( explainab*[tiab] OR interpretab*[tiab] OR accountab*[tiab] OR trust*[tiab]  )  AND  (  "machine learning"[tiab] OR "deep learning"[tiab] OR "artificial intelligence"[tiab]  OR machine learning[MeSH Terms] OR deep learning[MeSH Terms]  )  AND  (  cancer[MeSH Terms] OR cancer[tiab]  )  NOT review[Publication Type]  NOT meta-analysis[Publication Type]  NOT systematic review[Publication Type]  AND English[Language] |

**Scopus query (Elsevier)**

| **Platform/interface** | Elsevier |
| --- | --- |
| Search execution dates | Initial search:  Updated search: |
| Coverage intended | 2017-2024 |
| Limits applied | English only; exclusion of review articles (DOCTYPE re) |
| Query | (  TITLE-ABS-KEY(explainab*)  OR TITLE-ABS-KEY(interpretab*)  OR TITLE-ABS(accountab*)  OR TITLE-ABS(trust*)  )  AND  (  TITLE-ABS-KEY("machine learning")  OR TITLE-ABS-KEY("artificial intelligence")  OR TITLE-ABS-KEY("deep learning")  )  AND TITLE-ABS-KEY(cancer)  AND NOT DOCTYPE(re)  AND LANGUAGE(english) |
