# Supplementary material for: Explainable AI in Cancer Imaging: Scoping Review of Methods, Modalities, and Clinical Integration
Source: J Med Internet Res. 2026 May 20;28:e80645. doi: 10.2196/80645 (PMC13189567; doi:10.2196/80645)
Supplement: Multimedia Appendix 2 [file jmir-v28-e80645-s002.docx]

Data extraction schema used in the review

**Supplementary Table S1.** Overview of data fields used for information extraction from each study.

|  | Field | Meaning |
| --- | --- | --- |
|  |  |  |
| **Study Identification** |  |  |
|  | Title | The title of the study |
|  | Authors | Names of the study’s authors |
|  | Year | The year the study was published |
|  | Source | Publishing venue |
| **Study Characteristics** |  |  |
|  | Cancer | Type of cancer and affected organ |
|  | Study Aim | Primary objective of the study |
|  | Dataset | Openness and origin of dataset |
|  | N Participants | The total count of participants or records used in the study |
| **Data methods** |  |  |
|  | Data Preprocessing | Preprocessing of imaging techniques employed in the study |
|  | Other Types of Data | Additional types of data used in the study |
|  | Imaging Modality | Types of imaging techniques used |
|  | Features | Types of features used for training the algorithms |
|  | Feature Selection | Methods used to select relevant features for the analysis |
|  | ML Goal | The goal of the machine learning model, such as prognosis, diagnosis, or treatment proposal |
| **AI / Machine Learning aspects** |  |  |
|  | ML/DL Method | Specific machine learning or deep learning methods used in the study |
|  | (Cross-) Validation Method | The procedure used for validation, such as cross-validation |
|  | Technical Validation | Methods used for technical validation, such as using an external test set |
| **Clinical aspects** |  |  |
|  | ML Performance | Evaluation metrics and clinical performance |
|  | Clinical Validation | Whether a pilot or randomized controlled trial (RCT) was conducted for clinical validation |
| **Explainability and xAI** |  |  |
|  | Clinical Performance Metric Used in xAI Validation | Performance results from clinical validation specific to the XAI |
|  | xAI Validation Users | Whether validation of the XAI was conducted (Yes/No) |
|  | Users Involved in xAI Validation | Whether users participated in the validation of the xAI |
|  | User Roles in xAI Validation | The specific user roles involved in the XAI validation |
|  | xAI Output Details | Details provided by XAI (e.g., heatmaps) |
|  | xAI Terms | The type of information offered by the XAI technique (e.g., image, terms like explainable, trust) |
|  | XAI Aim | The specific purpose for using explainable AI (XAI) |
|  | Type of XAI Method | Classification of the XAI method (e.g., post-hoc, transparent) |
|  | XAI Method | Methods used to explain the model |
| **Outcomes and Limitations** |  |  |
|  | Secondary and Outcome (Primary) | Any additional, optional outcomes or main outcomes |
|  | Limitations | Limitations identified |
|  | Code included | Whether code is provided |
