# Supplementary material for: Explainable AI in Cancer Imaging: Scoping Review of Methods, Modalities, and Clinical Integration
Source: J Med Internet Res. 2026 May 20;28:e80645. doi: 10.2196/80645 (PMC13189567; doi:10.2196/80645)
Supplement: Multimedia Appendix 3 [file jmir-v28-e80645-s003.docx]

1. Shen Y, Wu N, Phang J, Park J, Liu K, Tyagi S, et al. An interpretable classifier for high-resolution breast cancer screening images utilizing weakly supervised localization. Medical Image Analysis. 2021;68:101908. doi:10.1016/j.media.2020.101908 PMID: 33383334

2. Li J, Wang P, Zhou Y, Liang H, Luan K. Different Machine Learning and Deep Learning Methods for the Classification of Colorectal Cancer Lymph Node Metastasis Images. Front. Bioeng. Biotechnol.. 2021;8. doi:10.3389/fbioe.2020.620257 PMID: 33520971

3. Han F, Liao S, Yuan S, Wu R, Zhao Y, Xie Y. Explainable Prediction Of Renal Cell Carcinoma From Contrast-Enhanced CT Images Using Deep Convolutional Transfer Learning And The Shapley Additive Explanations Approach. 2021 IEEE International Conference on Image Processing (ICIP). 2021:3802-3806. doi:10.1109/icip42928.2021.9506144

4. Chen D, Zhao H, He J, Pan Q, Zhao W. An Causal XAI Diagnostic Model for Breast Cancer Based on Mammography Reports. 2021 IEEE International Conference on Bioinformatics and Biomedicine (BIBM). 2021:3341-3349. doi:10.1109/bibm52615.2021.9669648

5. Zhang B, Vakanski A, Xian M. Bi-Rads-Net: An Explainable Multitask Learning Approach for Cancer Diagnosis in Breast Ultrasound Images. 2021 IEEE 31st International Workshop on Machine Learning for Signal Processing (MLSP). 2021:1-6. doi:10.1109/mlsp52302.2021.9596314 PMID: 35509454

6. Zhang X, Wang K, Zhang X, Huang S. Pulmonary Nodule Classification of CT Images with Attribute Self-guided Graph Convolutional V-Shape Networks. Lecture Notes in Computer Science. 2021:280-292. doi:10.1007/978-3-030-89188-6_21

7. Prezioso E, Izzo S, Giampaolo F, Piccialli F, Dell'Aversana Orabona G, Cuocolo R, et al. Predictive Medicine for Salivary Gland Tumours Identification Through Deep Learning. IEEE J. Biomed. Health Inform.. 2022;26(10):4869-4879. doi:10.1109/jbhi.2021.3120178 PMID: 34648462

8. Kaliyugarasan S, Lundervold A, Selvikvåg Lundervold A. Pulmonary Nodule Classification in Lung Cancer from 3D Thoracic CT Scans Using fastai and MONAI.. IJIMAI. 2021;6(7):83-89. doi:10.9781/ijimai.2021.05.002

9. Chen D, Zhong K, He J. BDCN: Semantic Embedding Self-explanatory Breast Diagnostic Capsules Network. Lecture Notes in Computer Science. 2021:419-433. doi:10.1007/978-3-030-84186-7_28

10. Mukherjee P, Pal M, Ghosh L, Konar A. A Generative Model Based Approach for Zero-Shot Breast Cancer Segmentation Explaining Pixels’ Contribution to the Model’s Prediction. Studies in Computational Intelligence. 2021:401-425. doi:10.1007/978-3-030-64949-4_13

11. Gulum MA, Trombley CM, Kantardzic M. Multiple Interpretations Improve Deep Learning Transparency for Prostate Lesion Detection. Lecture Notes in Computer Science. 2021:120-137. doi:10.1007/978-3-030-71055-2_11 PMID: 28118817

12. An F, Li X, Ma X. Medical Image Classification Algorithm Based on Visual Attention Mechanism‐MCNN. Oxidative Medicine and Cellular Longevity. 2021;2021(1). doi:10.1155/2021/6280690 PMID: 33688390

13. Vuong D, Tanadini-Lang S, Wu Z, Marks R, Unkelbach J, Hillinger S, et al. Radiomics Feature Activation Maps as a New Tool for Signature Interpretability. Front. Oncol.. 2020;10. doi:10.3389/fonc.2020.578895 PMID: 33364192

14. Shen T, Wang J, Gou C, Wang FY. Hierarchical Fused Model With Deep Learning and Type-2 Fuzzy Learning for Breast Cancer Diagnosis. IEEE Trans. Fuzzy Syst.. 2020;28(12):3204-3218. doi:10.1109/tfuzz.2020.3013681

15. van der Velden BHM, Janse MHA, Ragusi MAA, Loo CE, Gilhuijs KGA. Volumetric breast density estimation on MRI using explainable deep learning regression. Sci Rep. 2020;10(1). doi:10.1038/s41598-020-75167-6 PMID: 33093572

16. Akamine Y, Ueda Y, Ueno Y, Sofue K, Murakami T, Yoneyama M, et al. Application of hierarchical clustering to multi-parametric MR in prostate: Differentiation of tumor and normal tissue with high accuracy. Magnetic Resonance Imaging. 2020;74:90-95. doi:10.1016/j.mri.2020.09.011 PMID: 32926991

17. Li T, Fong S, Siu SW, Yang Xs, Liu LS, Mohammed S. White learning methodology: A case study of cancer-related disease factors analysis in real-time PACS environment. Computer Methods and Programs in Biomedicine. 2020;197:105724. doi:10.1016/j.cmpb.2020.105724 PMID: 32877817

18. Xu W, Wang K, Lin J, Lu Y, Huang S, Zhang X. Knowledge-Guided And Hyper-Attention Aware Joint Network For Benign-Malignant Lung Nodule Classification. 2020 IEEE International Conference on Image Processing (ICIP). 2020:310-314. doi:10.1109/icip40778.2020.9190653

19. Jiang H, Gao F, Xu X, Huang F, Zhu S. Attentive and ensemble 3D dual path networks for pulmonary nodules classification. Neurocomputing. 2020;398:422-430. doi:10.1016/j.neucom.2019.03.103

20. Pintelas E, Liaskos M, Livieris IE, Kotsiantis S, Pintelas P. Explainable Machine Learning Framework for Image Classification Problems: Case Study on Glioma Cancer Prediction. J. Imaging. 2020;6(6):37. doi:10.3390/jimaging6060037 PMID: 34460583

21. Li Y, Gu D, Wen Z, Jiang F, Liu S. Classify and Explain: An Interpretable Convolutional Neural Network For Lung Cancer Diagnosis. ICASSP 2020 - 2020 IEEE International Conference on Acoustics, Speech and Signal Processing (ICASSP). 2020:1065-1069. doi:10.1109/icassp40776.2020.9054605

22. Ness L, Barkan E, Ozery-Flato M. Improving the Performance and Explainability of Mammogram Classifiers with Local Annotations. Lecture Notes in Computer Science. 2020:33-42. doi:10.1007/978-3-030-61166-8_4

23. Patil R, Wee L, Dekker A. Auto Segmentation of Lung in Non-small Cell Lung Cancer Using Deep Convolution Neural Network. Communications in Computer and Information Science. 2020:340-351. doi:10.1007/978-981-15-6634-9_31

24. van der Velden BH., Ragusi MAA, Janse MHA, Loo CE, Gilhuijs KGA. Interpretable deep learning regression for breast density estimation on MRI. Medical Imaging 2020: Computer-Aided Diagnosis. 2020:69. doi:10.1117/12.2549003

25. Li H, Peng X, Zeng J, Xiao J, Nie D, Zu C, et al. Explainable attention guided adversarial deep network for 3D radiotherapy dose distribution prediction. Knowledge-Based Systems. 2022;241:108324. doi:10.1016/j.knosys.2022.108324

26. Wang J, Jiang J, Zhang D, Zhang Yz, Guo L, Jiang Y, et al. An integrated AI model to improve diagnostic accuracy of ultrasound and output known risk features in suspicious thyroid nodules. Eur Radiol. 2022;32(3):2120-2129. doi:10.1007/s00330-021-08298-7 PMID: 34657970

27. Ma M, Liu R, Wen C, Xu W, Xu Z, Wang S, et al. Predicting the molecular subtype of breast cancer and identifying interpretable imaging features using machine learning algorithms. Eur Radiol. 2022;32(3):1652-1662. doi:10.1007/s00330-021-08271-4 PMID: 34647174

28. He W, Li B, Liao R, Mo H, Tian L. An ISHAP-based interpretation-model-guided classification method for malignant pulmonary nodule. Knowledge-Based Systems. 2022;237:107778. doi:10.1016/j.knosys.2021.107778

29. Wu X, Li M, Cui Xw, Xu G. Deep multimodal learning for lymph node metastasis prediction of primary thyroid cancer. Phys. Med. Biol.. 2022;67(3):035008. doi:10.1088/1361-6560/ac4c47 PMID: 35042207

30. Kobylińska K, Orłowski T, Adamek M, Biecek P. Explainable Machine Learning for Lung Cancer Screening Models. Applied Sciences. 2022; 12(4):1926. https://doi.org/10.3390/app12041926

31. Hassan MR, Islam MF, Uddin MZ, Ghoshal G, Hassan MM, Huda S, et al. Prostate cancer classification from ultrasound and MRI images using deep learning based Explainable Artificial Intelligence. Future Generation Computer Systems. 2022;127:462-472. doi:10.1016/j.future.2021.09.030

32. Chaunzwa TL, Hosny A, Xu Y, Shafer A, Diao N, Lanuti M, et al. Deep learning classification of lung cancer histology using CT images. Sci Rep. 2021;11(1). doi:10.1038/s41598-021-84630-x PMID: 33727623

33. Su R, Liu X, Jin Q, Liu X, Wei L. Identification of glioblastoma molecular subtype and prognosis based on deep MRI features. Knowledge-Based Systems. 2021;232:107490. doi:10.1016/j.knosys.2021.107490

34. Lee IC, Huang JY, Chen TC, Yen CH, Chiu NC, Hwang HE, et al. Evolutionary Learning-Derived Clinical-Radiomic Models for Predicting Early Recurrence of Hepatocellular Carcinoma after Resection. Liver Cancer. 2021;10(6):572-582. doi:10.1159/000518728 PMID: 34950180

35. Horry M, Chakraborty S, Pradhan B, Paul M, Gomes D, Ul-Haq A, et al. Deep Mining Generation of Lung Cancer Malignancy Models from Chest X-ray Images. Sensors. 2021;21(19):6655. doi:10.3390/s21196655 PMID: 34640976

36. Tardy M, Mateus D. Looking for Abnormalities in Mammograms With Self- and Weakly Supervised Reconstruction. IEEE Trans. Med. Imaging. 2021;40(10):2711-2722. doi:10.1109/tmi.2021.3050040 PMID: 33417539

37. Chen X, Li Y, Li X, Cao X, Xiang Y, Xia W, et al. An interpretable machine learning prognostic system for locoregionally advanced nasopharyngeal carcinoma based on tumor burden features. Oral Oncology. 2021;118:105335. doi:10.1016/j.oraloncology.2021.105335 PMID: 34023742

38. Smedley NF, Aberle DR, Hsu W. Using deep neural networks and interpretability methods to identify gene expression patterns that predict radiomic features and histology in non-small cell lung cancer. J. Med. Imag.. 2021;8(03). doi:10.1117/1.jmi.8.3.031906 PMID: 33977113

39. Han L, Kamdar MR. MRI to MGMT: predicting methylation status in glioblastoma patients using convolutional recurrent neural networks. Biocomputing 2018. 2018:331-342. doi:10.1142/9789813235533_0031

40. Pota M, Scalco E, Sanguineti G, Farneti A, Cattaneo GM, Rizzo G, et al. Early prediction of radiotherapy-induced parotid shrinkage and toxicity based on CT radiomics and fuzzy classification. Artificial Intelligence in Medicine. 2017;81:41-53. doi:10.1016/j.artmed.2017.03.004 PMID: 28325604

41. Choi KS, Choi SH, Jeong B. Prediction of IDH genotype in gliomas with dynamic susceptibility contrast perfusion MR imaging using an explainable recurrent neural network. Neuro-Oncology. 2019;21(9):1197-1209. doi:10.1093/neuonc/noz095 PMID: 31127834

42. Paul R, Schabath M, Balagurunathan Y, Liu Y, Li Q, Gillies R, et al. Explaining Deep Features Using Radiologist-Defined Semantic Features and Traditional Quantitative Features. Tomography. 2019;5(1):192-200. doi:10.18383/j.tom.2018.00034 PMID: 30854457

43. Wang CJ, Hamm CA, Savic LJ, Ferrante M, Schobert I, Schlachter T, et al. Deep learning for liver tumor diagnosis part II: convolutional neural network interpretation using radiologic imaging features. Eur Radiol. 2019;29(7):3348-3357. doi:10.1007/s00330-019-06214-8 PMID: 31093705

44. Afshar P, Plataniotis KN, Mohammadi A. Capsule Networks’ Interpretability for Brain Tumor Classification Via Radiomics Analyses. 2019 IEEE International Conference on Image Processing (ICIP). 2019:3816-3820. doi:10.1109/icip.2019.8803615

45. Goncalves A, Ray P, Soper B, Widemann D, Nygård M, Nygård JF, et al. Bayesian multitask learning regression for heterogeneous patient cohorts. Journal of Biomedical Informatics. 2019;100:100059. doi:10.1016/j.yjbinx.2019.100059 PMID: 34384572

46. Shen S, Han SX, Aberle DR, Bui AA, Hsu W. An interpretable deep hierarchical semantic convolutional neural network for lung nodule malignancy classification. Expert Systems with Applications. 2019;128:84-95. doi:10.1016/j.eswa.2019.01.048 PMID: 31296975

47. Verma, Abhishek & Abhishek, & Verma, Shekhar. (2019). An Interpretable SVM Based Model for Cancer Prediction in Mammograms: First International Conference, CNC 2018, Gwalior, India, March 22-24, 2018, Revised Selected Papers. 10.1007/978-981-13-2372-0_39

48. Giraud P, Giraud P, Nicolas E, Boisselier P, Alfonsi M, Rives M, et al. Interpretable Machine Learning Model for Locoregional Relapse Prediction in Oropharyngeal Cancers. Cancers. 2020;13(1):57. doi:10.3390/cancers13010057 PMID: 33379188

49. Windisch P, Weber P, Fürweger C, Ehret F, Kufeld M, Zwahlen D, et al. Implementation of model explainability for a basic brain tumor detection using convolutional neural networks on MRI slices. Neuroradiology. 2020;62(11):1515-1518. doi:10.1007/s00234-020-02465-1 PMID: 32500277

50. Sanyal J, Banerjee I, Hahn L, Rubin D. An Automated Two-step Pipeline for Aggressive Prostate Lesion Detection from Multi-parametric MR Sequence.. AMIA Jt Summits Transl Sci Proc. 2020;2020:552-560. PMID: 32477677

51. Wang W, Charkborty G. Automatic prognosis of lung cancer using heterogeneous deep learning models for nodule detection and eliciting its morphological features. Appl Intell. 2021;51(4):2471-2484. doi:10.1007/s10489-020-01990-z

52. Pan D, Liu R, Zheng B, Yuan J, Zeng H, He Z, et al. Using Machine Learning to Unravel the Value of Radiographic Features for the Classification of Bone Tumors. BioMed Research International. 2021;2021(1). doi:10.1155/2021/8811056 PMID: 33791381

53. Cho Hh, Lee HY, Kim E, Lee G, Kim J, Kwon J, et al. Radiomics-guided deep neural networks stratify lung adenocarcinoma prognosis from CT scans. Commun Biol. 2021;4(1). doi:10.1038/s42003-021-02814-7 PMID: 34773070

54. Shim KY, Chung SW, Jeong JH, Hwang I, Park CK, Kim TM, et al. Radiomics-based neural network predicts recurrence patterns in glioblastoma using dynamic susceptibility contrast-enhanced MRI. Sci Rep. 2021;11(1). doi:10.1038/s41598-021-89218-z PMID: 33976264

55. Liu SC, Lai J, Huang JY, Cho CF, Lee PH, Lu MH, et al. Predicting microvascular invasion in hepatocellular carcinoma: a deep learning model validated across hospitals. Cancer Imaging. 2021;21(1). doi:10.1186/s40644-021-00425-3 PMID: 34627393

56. Radebe L, van der Kaay DCM, Wasserman JD, Goldenberg A. Predicting Malignancy in Pediatric Thyroid Nodules: Early Experience With Machine Learning for Clinical Decision Support. The Journal of Clinical Endocrinology &amp; Metabolism. 2021;106(12):e5236-e5246. doi:10.1210/clinem/dgab435 PMID: 34160618

57. Dong F, She R, Cui C, Shi S, Hu X, Zeng J, et al. One step further into the blackbox: a pilot study of how to build more confidence around an AI-based decision system of breast nodule assessment in 2D ultrasound. Eur Radiol. 2021;31(7):4991-5000. doi:10.1007/s00330-020-07561-7 PMID: 33404698

58. Jiang B, Zhang Y, Zhang L, H. de Bock G, Vliegenthart R, Xie X. Human-recognizable CT image features of subsolid lung nodules associated with diagnosis and classification by convolutional neural networks. Eur Radiol. 2021;31(10):7303-7315. doi:10.1007/s00330-021-07901-1 PMID: 33847813

59. Morgan HE, Wang K, Dohopolski M, Liang X, Folkert MR, Sher DJ, et al. Exploratory ensemble interpretable model for predicting local failure in head and neck cancer: the additive benefit of CT and intra-treatment cone-beam computed tomography features. Quant Imaging Med Surg. 2021;11(12):4781-4796. doi:10.21037/qims-21-274 PMID: 34888189

60. Wei L, Owen D, Rosen B, Guo X, Cuneo K, Lawrence TS, et al. A deep survival interpretable radiomics model of hepatocellular carcinoma patients. Physica Medica. 2021;82:295-305. doi:10.1016/j.ejmp.2021.02.013 PMID: 33714190

61. Zhao X, Wang X, Xia W, Zhang R, Jian J, Zhang J, et al. 3D multi-scale, multi-task, and multi-label deep learning for prediction of lymph node metastasis in T1 lung adenocarcinoma patients' CT images. Computerized Medical Imaging and Graphics. 2021;93:101987. doi:10.1016/j.compmedimag.2021.101987 PMID: 34610501

62. Gaur L, Bhandari M, Razdan T, Mallik S, Zhao Z. Explanation-Driven Deep Learning Model for Prediction of Brain Tumour Status Using MRI Image Data. Front. Genet.. 2022;13. doi:10.3389/fgene.2022.822666 PMID: 35360838

63. Kim ST, Lee JH, Lee H, Ro YM. Visually interpretable deep network for diagnosis of breast masses on mammograms. Phys. Med. Biol.. 2018;63(23):235025. doi:10.1088/1361-6560/aaef0a PMID: 30511660

64. Laios A, Kalampokis E, Johnson R, Thangavelu A, Tarabanis C, Nugent D, et al. Explainable Artificial Intelligence for Prediction of Complete Surgical Cytoreduction in Advanced-Stage Epithelial Ovarian Cancer. JPM. 2022;12(4):607. doi:10.3390/jpm12040607 PMID: 35455723

65. Zeineldin RA, Karar ME, Elshaer Z, Coburger ·, Wirtz CR, Burgert O, et al. Explainability of deep neural networks for MRI analysis of brain tumors. Int J CARS. 2022;17(9):1673-1683. doi:10.1007/s11548-022-02619-x PMID: 35460019

66. Han X, Chang L, Song K, Cheng L, Li M, Wei X. Multitask network for thyroid nodule diagnosis based on TI‐RADS. Medical Physics. 2022;49(8):5064-5080. doi:10.1002/mp.15724 PMID: 35608232

67. Yao MMS, Du H, Hartman M, Chan WP, Feng M. End-to-End Calcification Distribution Pattern Recognition for Mammograms: An Interpretable Approach with GNN. Diagnostics. 2022;12(6):1376. doi:10.3390/diagnostics12061376 PMID: 35741186

68. Islam MN, Hasan M, Hossain MK, Alam MGR, Uddin MZ, Soylu A. Vision transformer and explainable transfer learning models for auto detection of kidney cyst, stone and tumor from CT-radiography. Sci Rep. 2022;12(1). doi:10.1038/s41598-022-15634-4 PMID: 35794172

69. Manh VT, Zhou J, Jia X, Lin Z, Xu W, Mei Z, et al. Multi-Attribute Attention Network for Interpretable Diagnosis of Thyroid Nodules in Ultrasound Images. IEEE Trans. Ultrason., Ferroelect., Freq. Contr.. 2022;69(9):2611-2620. doi:10.1109/tuffc.2022.3190012 PMID: 35820014

70. Severn C, Suresh K, Görg C, Choi YS, Jain R, Ghosh D. A Pipeline for the Implementation and Visualization of Explainable Machine Learning for Medical Imaging Using Radiomics Features. Sensors. 2022;22(14):5205. doi:10.3390/s22145205 PMID: 35890885

71. Zou Y, Shi Y, Sun F, Liu J, Guo Y, Zhang H, et al. Extreme gradient boosting model to assess risk of central cervical lymph node metastasis in patients with papillary thyroid carcinoma: Individual prediction using SHapley Additive exPlanations. Computer Methods and Programs in Biomedicine. 2022;225:107038. doi:10.1016/j.cmpb.2022.107038 PMID: 35930861

72. Maqsood S, Damaševičius R, Maskeliūnas R. Multi-Modal Brain Tumor Detection Using Deep Neural Network and Multiclass SVM. Medicina. 2022;58(8):1090. doi:10.3390/medicina58081090 PMID: 36013557

73. Jin Z, Pei S, Ouyang L, Zhang L, Mo X, Chen Q, et al. Thy‐Wise: An interpretable machine learning model for the evaluation of thyroid nodules. Intl Journal of Cancer. 2022;151(12):2229-2243. doi:10.1002/ijc.34248 PMID: 36095154

74. Chen K, Cao J, Zhang X, Wang X, Zhao X, Li Q, et al. Differentiation between spinal multiple myeloma and metastases originated from lung using multi-view attention-guided network. Front. Oncol.. 2022;12. doi:10.3389/fonc.2022.981769 PMID: 36158659

75. Shao L, Liu Z, Liu J, Yan Y, Sun K, Liu X, et al. Patient-level grading prediction of prostate cancer from mp-MRI via GMINet. Computers in Biology and Medicine. 2022;150:106168. doi:10.1016/j.compbiomed.2022.106168 PMID: 36240594

76. Deng P, Han X, Wei X, Chang L. Automatic classification of thyroid nodules in ultrasound images using a multi-task attention network guided by clinical knowledge. Computers in Biology and Medicine. 2022;150:106172. doi:10.1016/j.compbiomed.2022.106172 PMID: 36242812

77. Duan J, Zhao Y, Sun Q, Liang D, Liu Z, Chen X, et al. Imaging‐proteomic analysis for prediction of neoadjuvant chemotherapy responses in patients with breast cancer. Cancer Medicine. 2023;12(23):21256-21269. doi:10.1002/cam4.6704 PMID: 37962087

78. Saber R, Henault D, Messaoudi N, Rebolledo R, Montagnon E, Soucy G, et al. Radiomics using computed tomography to predict CD73 expression and prognosis of colorectal cancer liver metastases. J Transl Med. 2023;21(1). doi:10.1186/s12967-023-04175-7 PMID: 37501197

79. Ibrahim A, Vaidyanathan A, Primakov S, Belmans F, Bottari F, Refaee T, et al. Deep learning based identification of bone scintigraphies containing metastatic bone disease foci. Cancer Imaging. 2023;23(1). doi:10.1186/s40644-023-00524-3 PMID: 36698217

80. Visonà G, Spiller LM, Hahn S, Hattingen E, Vogl TJ, Schweikert G, et al. Machine-Learning-Aided Prediction of Brain Metastases Development in Non–Small-Cell Lung Cancers. Clinical Lung Cancer. 2023;24(8):e311-e322. doi:10.1016/j.cllc.2023.08.002 PMID: 37689579

81. Kang S, Chen Z, Li L, Lu W, Qi XS, Tan S. Learning feature fusion via an interpretation method for tumor segmentation on PET/CT. Applied Soft Computing. 2023;148:110825. doi:10.1016/j.asoc.2023.110825

82. Dong Y, Li X, Yang Y, Wang M, Gao B. A Synthesizing Semantic Characteristics Lung Nodules Classification Method Based on 3D Convolutional Neural Network. Bioengineering. 2023;10(11):1245. doi:10.3390/bioengineering10111245 PMID: 38002369

83. Xiong L, Chen C, Lin Y, Mao W, Song Z. A computer-aided determining method for the myometrial infiltration depth of early endometrial cancer on MRI images. BioMed Eng OnLine. 2023;22(1). doi:10.1186/s12938-023-01169-w PMID: 37907955

84. Li M, Zhou H, Li X, Yan P, Jiang Y, Luo H, et al. SDA-Net: Self-distillation driven deformable attentive aggregation network for thyroid nodule identification in ultrasound images. Artificial Intelligence in Medicine. 2023;146:102699. doi:10.1016/j.artmed.2023.102699 PMID: 38042598

85. Jiang Y, Zhang Z, Wang W, Huang W, Chen C, Xi S, et al. Biology-guided deep learning predicts prognosis and cancer immunotherapy response. Nat Commun. 2023;14(1). doi:10.1038/s41467-023-40890-x PMID: 37612313

86. Saeed N, Ridzuan M, Alasmawi H, Sobirov I, Yaqub M. MGMT promoter methylation status prediction using MRI scans? An extensive experimental evaluation of deep learning models. Medical Image Analysis. 2023;90:102989. doi:10.1016/j.media.2023.102989 PMID: 37827111

87. Verma A, Gupta N, Bhatele P, Khanna P. JMCD Dataset for Brain Tumor Detection and Analysis Using Explainable Deep Learning. SN COMPUT. SCI.. 2023;4(6). doi:10.1007/s42979-023-02308-9

88. Orton MR, Hann E, Doran SJ, Shepherd STC, Ap Dafydd D, Spencer CE, et al. Interpretability of radiomics models is improved when using feature group selection strategies for predicting molecular and clinical targets in clear-cell renal cell carcinoma: insights from the TRACERx Renal study. Cancer Imaging. 2023;23(1). doi:10.1186/s40644-023-00594-3 PMID: 37580840

89. Jin W, Shen L, Tian Y, Zhu H, Zou N, Zhang M, et al. Improving the prediction of Spreading Through Air Spaces (STAS) in primary lung cancer with a dynamic dual-delta hybrid machine learning model: a multicenter cohort study. Biomark Res. 2023;11(1). doi:10.1186/s40364-023-00539-9 PMID: 37996894

90. Yu Y, Ren W, He Z, Chen Y, Tan Y, Mao L, et al. Machine learning radiomics of magnetic resonance imaging predicts recurrence-free survival after surgery and correlation of LncRNAs in patients with breast cancer: a multicenter cohort study. Breast Cancer Res. 2023;25(1). doi:10.1186/s13058-023-01688-3 PMID: 37915093

91. Fan M, Huang G, Lou J, Gao X, Zeng T, Li L. Cross-Parametric Generative Adversarial Network-Based Magnetic Resonance Image Feature Synthesis for Breast Lesion Classification. IEEE J. Biomed. Health Inform.. 2023;27(11):5495-5505. doi:10.1109/jbhi.2023.3311021 PMID: 37656652

92. Bouzar-Benlabiod L, Harrar K, Yamoun L, Khodja MY, Akhloufi MA. A novel breast cancer detection architecture based on a CNN-CBR system for mammogram classification. Computers in Biology and Medicine. 2023;163:107133. doi:10.1016/j.compbiomed.2023.107133 PMID: 37327756

93. Deng F, Liu Z, Fang W, Niu L, Chu X, Cheng Q, et al. MRI radiomics for brain metastasis sub-pathology classification from non-small cell lung cancer: a machine learning, multicenter study. Phys Eng Sci Med. 2023;46(3):1309-1320. doi:10.1007/s13246-023-01300-0 PMID: 37460894

94. Mercaldo F, Brunese L, Martinelli F, Santone A, Cesarelli M. Explainable Convolutional Neural Networks for Brain Cancer Detection and Localisation. Sensors. 2023;23(17):7614. doi:10.3390/s23177614 PMID: 37688069

95. Pan X, Feng T, Liu C, Savjani RR, Chin RK, Sharon Qi X. A survival prediction model via interpretable machine learning for patients with oropharyngeal cancer following radiotherapy. J Cancer Res Clin Oncol. 2023;149(10):6813-6825. doi:10.1007/s00432-023-04644-y PMID: 36807760

96. Li J, Fan X, Tang T, Wu E, Wang D, Zong H, et al. An artificial intelligence method for predicting postoperative urinary incontinence based on multiple anatomic parameters of MRI. Heliyon. 2023;9(10):e20337. doi:10.1016/j.heliyon.2023.e20337 PMID: 37767466

97. Li Z, Jiang Y, Lu M, Li R, Xia Y. Survival Prediction via Hierarchical Multimodal Co-Attention Transformer: A Computational Histology-Radiology Solution. IEEE Trans. Med. Imaging. 2023;42(9):2678-2689. doi:10.1109/tmi.2023.3263010 PMID: 37030860

98. She Z, Marzullo A, Destito M, Spadea MF, Leone R, Anzalone N, et al. Deep learning-based overall survival prediction model in patients with rare cancer: a case study for primary central nervous system lymphoma. Int J CARS. 2023;18(10):1849-1856. doi:10.1007/s11548-023-02886-2 PMID: 37083973

99. Hung SC, Wang YT, Tseng MH. An Interpretable Three-Dimensional Artificial Intelligence Model for Computer-Aided Diagnosis of Lung Nodules in Computed Tomography Images. Cancers. 2023;15(18):4655. doi:10.3390/cancers15184655 PMID: 37760624

100. Cao Y, Kunaprayoon D, Ren L. Interpretable AI-assisted clinical decision making (CDM) for dose prescription in radiosurgery of brain metastases. Radiotherapy and Oncology. 2023;187:109842. doi:10.1016/j.radonc.2023.109842 PMID: 37543055

101. Sun Z, Wang W, Huang W, Zhang T, Chen C, Yuan Q, et al. Noninvasive imaging evaluation of peritoneal recurrence and chemotherapy benefit in gastric cancer after gastrectomy：a multicenter study. International Journal of Surgery. 2023;Publish Ahead of Print. doi:10.1097/js9.0000000000000328 PMID: 37300884

102. Bashkanov O, Rak M, Meyer A, Engelage L, Lumiani A, Muschter R, et al. Automatic detection of prostate cancer grades and chronic prostatitis in biparametric MRI. Computer Methods and Programs in Biomedicine. 2023;239:107624. doi:10.1016/j.cmpb.2023.107624 PMID: 37271051

103. Sluckin TC, Hekhuis M, Kol SQ, Nederend J, Horsthuis K, Beets-Tan RGH, et al. A Deep Learning Framework with Explainability for the Prediction of Lateral Locoregional Recurrences in Rectal Cancer Patients with Suspicious Lateral Lymph Nodes. Diagnostics. 2023;13(19):3099. doi:10.3390/diagnostics13193099 PMID: 37835842

104. Salahuddin Z, Chen Y, Zhong X, Woodruff HC, Rad NM, Mali SA, et al. From Head and Neck Tumour and Lymph Node Segmentation to Survival Prediction on PET/CT: An End-to-End Framework Featuring Uncertainty, Fairness, and Multi-Region Multi-Modal Radiomics. Cancers. 2023;15(7):1932. doi:10.3390/cancers15071932 PMID: 37046593

105. Gerbasi A, Clementi G, Corsi F, Albasini S, Malovini A, Quaglini S, et al. DeepMiCa: Automatic Segmentation and Classification of Breast Microcalcifications from Mammograms. SSRN Journal. doi:10.2139/ssrn.4173901

106. Hamm CA, Baumgärtner GL, Biessmann F, Beetz NL, Hartenstein A, Savic LJ, et al. Interactive Explainable Deep Learning Model Informs Prostate Cancer Diagnosis at MRI. Radiology. 2023;307(4). doi:10.1148/radiol.222276 PMID: 37039688

107. Mo Y, Han C, Liu Y, Liu M, Shi Z, Lin J, et al. HoVer-Trans: Anatomy-Aware HoVer-Transformer for ROI-Free Breast Cancer Diagnosis in Ultrasound Images. IEEE Trans. Med. Imaging. 2023;42(6):1696-1706. doi:10.1109/tmi.2023.3236011 PMID: 37018705

108. Deshmukh S, Behera BK, Mulay P, et al. Explainable quantum clustering method to model medical data. Knowledge-Based Systems. 2023;267:110413. doi:10.1016/j.knosys.2023.110413

109. Prodan M, Paraschiv E, Stanciu A. Applying Deep Learning Methods for Mammography Analysis and Breast Cancer Detection. Applied Sciences. 2023;13(7):4272. doi:10.3390/app13074272

110. Ru J, Lu B, Chen B, Shi J, Chen G, Wang M, et al. Attention guided neural ODE network for breast tumor segmentation in medical images. Computers in Biology and Medicine. 2023;159:106884. doi:10.1016/j.compbiomed.2023.106884 PMID: 37071938

111. Hu J, Gu X, Wang Z, Gu X. Mixture of calibrated networks for domain generalization in brain tumor segmentation. Knowledge-Based Systems. 2023;270:110520. doi:10.1016/j.knosys.2023.110520

112. Song D, Yao J, Jiang Y, Shi S, Cui C, Wang L, et al. A new xAI framework with feature explainability for tumors decision-making in Ultrasound data: comparing with Grad-CAM. Computer Methods and Programs in Biomedicine. 2023;235:107527. doi:10.1016/j.cmpb.2023.107527 PMID: 37086704

113. Bobowicz M, Rygusik M, Buler J, Buler R, Ferlin M, Kwasigroch A, et al. Attention-Based Deep Learning System for Classification of Breast Lesions—Multimodal, Weakly Supervised Approach. Cancers. 2023;15(10):2704. doi:10.3390/cancers15102704 PMID: 37345041

114. Brocki L, Chung NC. Integration of Radiomics and Tumor Biomarkers in Interpretable Machine Learning Models. Cancers. 2023;15(9):2459. doi:10.3390/cancers15092459 PMID: 37173930

115. Ma T, Zhang Y, Zhao M, Wang L, Wang H, Ye Z. A machine learning-based radiomics model for prediction of tumor mutation burden in gastric cancer. Front. Genet.. 2023;14. doi:10.3389/fgene.2023.1283090 PMID: 38028587

116. Pang J, Yang M, Li J, Zhong X, Shen X, Chen T, et al. Interpretable machine learning model based on the systemic inflammation response index and ultrasound features can predict central lymph node metastasis in cN0T1–T2 papillary thyroid carcinoma. Gland Surg. 2023;12(11):1485-1499. doi:10.21037/gs-23-349 PMID: 38107491

117. Damineni DH, Sekharamantry PK, Badugu R. An Adaptable Model for Medical Image Classification Using the Streamlined Attention Mechanism. Int. J. Onl. Eng.. 2023;19(16):93-110. doi:10.3991/ijoe.v19i16.44461

118. Li WB, Du ZC, Liu YJ, Gao JX, Wang JG, Dai Q, et al. Prediction of axillary lymph node metastasis in early breast cancer patients with ultrasonic videos based deep learning. Front. Oncol.. 2023;13. doi:10.3389/fonc.2023.1219838 PMID: 37719009

119. Wang J, Gao W, Lu M, Yao X, Yang D. Development of an interpretable machine learning model for Ki-67 prediction in breast cancer using intratumoral and peritumoral ultrasound radiomics features. Front. Oncol.. 2023;13. doi:10.3389/fonc.2023.1290313 PMID: 38044998

120. Wang J, Zheng Y, Ma J, Li X, Wang C, Gee J, et al. Information bottleneck-based interpretable multitask network for breast cancer classification and segmentation. Medical Image Analysis. 2023;83:102687. doi:10.1016/j.media.2022.102687 PMID: 36436356

121. Zhang H, Chen L, Gu X, Zhang M, Qin Y, Yao F, et al. Trustworthy learning with (un)sure annotation for lung nodule diagnosis with CT. Medical Image Analysis. 2023;83:102627. doi:10.1016/j.media.2022.102627 PMID: 36283199

122. Roest C, Kwee T, Saha A, Fütterer J, Yakar D, Huisman H. AI-assisted biparametric MRI surveillance of prostate cancer: feasibility study. Eur Radiol. 2022;33(1):89-96. doi:10.1007/s00330-022-09032-7 PMID: 35960339

123. Terunuma T, Sakae T, Hu Y, Takei H, Moriya S, Okumura T, et al. Explainability and controllability of patient‐specific deep learning with attention‐based augmentation for markerless image‐guided radiotherapy. Medical Physics. 2023;50(1):480-494. doi:10.1002/mp.16095 PMID: 36354286

124. Qi H, An Y, Hu X, Miao S, Li J. Explainable Machine Learning Explores Association Between Sarcopenia and Breast Cancer Distant Metastasis. IEEE Access. 2023;11:65725-65738. doi:10.1109/access.2023.3289403

125. Zhu Z, Wang S. ODET: Optimized Deep ELM-based Transfer Learning for Breast Cancer Explainable Detection. EAI Endorsed Scal Inf Syst. 2022:e7. doi:10.4108/eetsis.v9i6.1747

126. Zhang B, Vakanski A, Xian M. BI-RADS-NET-V2: A Composite Multi-Task Neural Network for Computer-Aided Diagnosis of Breast Cancer in Ultrasound Images With Semantic and Quantitative Explanations. IEEE Access. 2023;11:79480-79494. doi:10.1109/access.2023.3298569 PMID: 37608804

127. Yadav A, Nisha F, Coskunuzer B. Breast Cancer Detection with Topological Machine Learning. In: Proceedings of the 2023 10th International Conference on Biomedical and Bioinformatics Engineering. ACM; 2023:217-222. doi:10.1145/3637732.3637744

128. Monteleone M, Gennai S, Govoni P, Paganelli C. A novel explainable approach in radiomics pipeline for local recurrence prediction of lung cancer: a feasibility study exploiting high energy physics potential to evaluate the model. Proceedings of the 2023 10th International Conference on Bioinformatics Research and Applications. 2023:184-187. doi:10.1145/3632047.3632074

129. Koyyada SP, Singh TP. An AI Decision System to Predict Lung Nodules through Localization from Chest X-ray Images. 2023 9th International Conference on Signal Processing and Communication (ICSC). 2023:214-220. doi:10.1109/icsc60394.2023.10441301

130. Acharjya K, M M, S AJ. Accurate Breast Tumor Identification Using Cutting-Edge Deep Learning. 2023 International Conference on Recent Advances in Science and Engineering Technology (ICRASET). 2023:1-5. doi:10.1109/icraset59632.2023.10420410

131. Peng T, Wang C, Hu T, Mao H, Di G, Zhang L. Interactive Ultrasound Prostate Cancer Segmentation using Deep Learning with Principal Curve-based Fine-tuning. 2023 IEEE International Conference on Bioinformatics and Biomedicine (BIBM). 2023:2165-2168. doi:10.1109/bibm58861.2023.10385872

132. Sumithra B, Vallathan G, Raman Kumar M, Govindharaju K. Deep Learning for Accurate Chest Disease Classification: A CNN-Based Approach for Lung Cancer Subtypes and Normal Cells. 2023 International Conference on System, Computation, Automation and Networking (ICSCAN). 2023:1-7. doi:10.1109/icscan58655.2023.10394855

133. Farrag A, Gad G, Fadlullah ZM, Fouda MM. Mammogram Tumor Segmentation with Preserved Local Resolution: An Explainable AI System. GLOBECOM 2023 - 2023 IEEE Global Communications Conference. 2023:314-319. doi:10.1109/globecom54140.2023.10436915

134. Murad NY, Hasan MH, Azam MH, Yousuf N, Khalique SA. Explaining Deep Learning Decisions Via Fuzzy Inference System on Medical Images. 2023 IEEE 21st Student Conference on Research and Development (SCOReD). 2023:7-12. doi:10.1109/scored60679.2023.10563415

135. Mercaldo F, Brunese L, Martinelli F, Santone A, Cesarelli M. Explainable Convolutional Neural Networks for Brain Cancer Detection and Localisation.. Sensors (Basel). 2023;23(17). doi:10.1016/j.jpdc.2018.04.008 PMID: 37688069

136. Zaridis DI, Mylona E, Tachos NS, Kalantzopoulos C, Marias K, Tsiknakis M, et al. Transi-Net: An Explainable Deep Learning Model Ensemble For Prostate's Transition Zone Segmentation. 2023 IEEE 23rd International Conference on Bioinformatics and Bioengineering (BIBE). 2023:422-426. doi:10.1109/bibe60311.2023.00075

137. Devarajan HR, Balasubramanian S, Kumar Swarnkar S, Kumar P, Jallepalli VR. Deep Learning for Automated Detection of Lung Cancer from Medical Imaging Data. In: 2023 International Conference on Artificial Intelligence for Innovations in Healthcare Industries (ICAIIHI). IEEE; 2023:1-5. doi:10.1109/ICAIIHI57871.2023.10488962

138. Liu S, Wang S, Wang Q, Luo J. Classification of Benign and Malignant Pulmonary Nodules Based on Mixed Features. 2023 42nd Chinese Control Conference (CCC). 2023:8803-8808. doi:10.23919/ccc58697.2023.10240557

139. Rahman A, Karim MR, Chowdhury P, Hossain A, Islam MM. NeuroXAI++: An Efficient X-AI Intensive Brain Cancer Detection and Localization. 2023 International Conference on Next-Generation Computing, IoT and Machine Learning (NCIM). 2023:1-6. doi:10.1109/ncim59001.2023.10212818

140. B S, Bhargavi MS. An XAI Approach to Predictive Analytics of Pancreatic Cancer. In: 2023 International Conference on Information Technology (ICIT). IEEE; 2023:343-348. doi:10.1109/ICIT58056.2023.10225991

141. Karimzadeh M, Vakanski A, Xian M, Zhang B. Post-Hoc Explainability of BI-RADS Descriptors in a Multi-Task Framework for Breast Cancer Detection and Segmentation. 2023 IEEE 33rd International Workshop on Machine Learning for Signal Processing (MLSP). 2023:1-6. doi:10.1109/mlsp55844.2023.10286006 PMID: 38572141

142. Sun Q, Xie Z, Han J, Cai Y, Xu L, Ma J. Ultrasonic Spectral Information Promotes Specificity and Visualization of Breast Cancer in Deep Learning. 2023 IEEE International Ultrasonics Symposium (IUS). 2023:1-4. doi:10.1109/ius51837.2023.10308082

143. Duan J, Zhao Y, Zhang Z, Liang D, Li ZC, Liu Z, et al. Imaging-proteomics co-profiling reveals biologic pathways underlying prognostic MRI features. 2023 15th Biomedical Engineering International Conference (BMEiCON). 2023:1-5. doi:10.1109/bmeicon60347.2023.10321994

144. Ugalde J, Godoy E, Mellado D, Cavieres E, Carvajal B, Fernández C, et al. Torwards Trustworthy Machine Learning based systems: Evaluating breast cancer predictions interpretability using Human Centered Machine Learning and UX Techniques. Communications in Computer and Information Science. 2023:538-545. doi:10.1007/978-3-031-36004-6_73

145. Carloni G, Pachetti E, Colantonio S. Causality-Driven One-Shot Learning for Prostate Cancer Grading from MRI. 2023 IEEE/CVF International Conference on Computer Vision Workshops (ICCVW). 2023:2608-2616. doi:10.1109/iccvw60793.2023.00276

146. Jin S, Xu H, Dong Y, Hao X, Qin F, Wang R, et al. Multiple Instance Learning for Lymph Node Metastasis Prediction from Cervical Cancer MRI. 2023 IEEE 20th International Symposium on Biomedical Imaging (ISBI). 2023:1-4. doi:10.1109/isbi53787.2023.10230666

147. Saber R, Henault D, Rebolledo R, Turcotte S, Kadoury S. Ensemble Tabnet Predicting a T-Cell/MHC-I-Based Immune Profile Biomarker for Colorectal Liver Metastases from CT Images. 2023 IEEE 20th International Symposium on Biomedical Imaging (ISBI). 2023:1-5. doi:10.1109/isbi53787.2023.10230665

148. La Greca Saint-Esteven A, Marchiori C, Bogowicz M, Barranco-García J, Khodabakhshi Z, Konukoglu E, et al. Diagnostic Accuracy and Reliability of Deep Learning-Based Human Papillomavirus Status Prediction in Oropharyngeal Cancer. Lecture Notes in Electrical Engineering. 2023:281-291. doi:10.1007/978-981-16-6775-6_23

149. B P, Gomathi R, Harshavardhana C, Reddy PK, Kumar D, Pundir AKS. Integrating Deep Learning and Graph Neural Networks for Multimodal Lung Tumor Analysis: A Novel Approach for Improved Classification and Predict. 2023 International Conference on Self Sustainable Artificial Intelligence Systems (ICSSAS). 2023:346-352. doi:10.1109/icssas57918.2023.10331691

150. Alomar A, Alazzam M, Mustafa H, Mustafa A. Lung Cancer Detection Using Deep Learning and Explainable Methods. In: 2023 14th International Conference on Information and Communication Systems (ICICS). IEEE; 2023:1-4. doi:10.1109/ICICS60529.2023.10330443

151. Contreras V, Bagante A, Marini N, Schumacher M, Andrearczyk V, Calvaresi D. Explanation Generation via Decompositional Rules Extraction for Head and Neck Cancer Classification. Lecture Notes in Computer Science. 2023:187-211. doi:10.1007/978-3-031-40878-6_11 PMID: 28118817

152. Ji C, Du C, Zhang Q, Wang S, Ma C, Xie J, et al. Mammo-Net: Integrating Gaze Supervision and Interactive Information in Multi-view Mammogram Classification. Lecture Notes in Computer Science. 2023:68-78. doi:10.1007/978-3-031-43990-2_7

153. Samaras AD, Apostolopoulos ID, Moustakidis S, Papageorgiou E, Papathanasiou ND, Apostolopoulos DJ, et al. Explainable Classification for Non-Small Cell Lung Cancer Based on Positron Emission Tomography Features and Clinical Data. 2023 14th International Conference on Information, Intelligence, Systems &amp; Applications (IISA). 2023:1-8. doi:10.1109/iisa59645.2023.10345893

154. Mellado D, Querales M, Sotelo J, Godoy E, Pardo F, Lever S, et al. A Deep Learning Classifier Using Sliding Patches For Detection of Mammographical Findings. 2023 19th International Symposium on Medical Information Processing and Analysis (SIPAIM). 2023:1-5. doi:10.1109/sipaim56729.2023.10373511

155. Buler J, Buler R, Bobowicz M, Ferlin M, Rygusik M, Kwasigroch A, et al. Interpretable deep learning approach for classification of breast cancer - a comparative analysis of multiple instance learning models. 2023 27th International Conference on Methods and Models in Automation and Robotics (MMAR). 2023:105-110. doi:10.1109/mmar58394.2023.10242564

156. Al Noman A, Arif ASM. Brain Tumor Recognition from MRI Using Deep Learning with Data Balancing Methods and Its Explainability with AI. Lecture Notes in Networks and Systems. 2023:523-538. doi:10.1007/978-981-99-7093-3_35

157. Wang D, Wang Y, Wang Y, Liu L, Li J, Huang Q. Fusion of Human Cognitive Knowledge and Machine Inference for Breast Cancer Detection. In: 2023 International Conference on Advanced Robotics and Mechatronics (ICARM). IEEE; 2023:179-184. doi:10.1109/ICARM58088.2023.10218759

158. Addala V. BREAST AI: Low Cost, Explainable Artificial Intelligence Based App for Efficient Diagnosis of Breast Cancer in Developing Areas. 2023 IEEE 3rd International Conference on Electronic Communications, Internet of Things and Big Data (ICEIB). 2023:164-167. doi:10.1109/iceib57887.2023.10170357

159. La Ferla M, Montebello M, Seychell D. An XAI Approach to Deep Learning Models in the Detection of DCIS. In: Maglogiannis I, Iliadis L, Papaleonidas A, Chochliouros I, eds. Artificial Intelligence Applications and Innovations. AIAI 2023 IFIP WG 12.5 International Workshops. IFIP Advances in Information and Communication Technology, vol 677. Springer; 2023. doi:10.1007/978-3-031-34171-7_33

160. Pal M, Mistry S, De D. Interpretability Approaches of Explainable AI in Analyzing Features for Lung Cancer Detection. Lecture Notes in Networks and Systems. 2023:277-287. doi:10.1007/978-981-19-5191-6_23

161. Romanov S, Howell S, Harkness E, Bydder M, Evans DG, Squires S, et al. Artificial Intelligence for Image-Based Breast Cancer Risk Prediction Using Attention. Tomography. 2023;9(6):2103-2115. doi:10.3390/tomography9060165 PMID: 38133069

162. Maaliw RR, Soni M, Delos Santos MP, de Veluz MRD, Lagrazon PGG, Seño MP, et al. AWFCNET: An Attention-Aware Deep Learning Network with Fusion Classifier for Breast Cancer Classification Using Enhanced Mammograms. 2023 IEEE World AI IoT Congress (AIIoT). 2023:0736-0744. doi:10.1109/aiiot58121.2023.10174427

163. Ahmed S, Nobel SN, Ullah O. An Effective Deep CNN Model for Multiclass Brain Tumor Detection Using MRI Images and SHAP Explainability. 2023 International Conference on Electrical, Computer and Communication Engineering (ECCE). 2023:1-6. doi:10.1109/ecce57851.2023.10101503

164. Ye JY, Fang P, Peng ZP, Huang XT, Xie JZ, Yin XY. A radiomics-based interpretable model to predict the pathological grade of pancreatic neuroendocrine tumors. Eur Radiol. 2023;34(3):1994-2005. doi:10.1007/s00330-023-10186-1 PMID: 37658884

165. Osadebey M, Liu Q, Fuster-Garcia E, Emblem KE. Interpreting deep learning models for glioma survival classification using visualization and textual explanations. BMC Med Inform Decis Mak. 2023;23(1). doi:10.1186/s12911-023-02320-2 PMID: 37853371

166. Zhang Y, Qu H, Tian Y, Na F, Yan J, Wu Y, et al. PB-LNet: a model for predicting pathological subtypes of pulmonary nodules on CT images. BMC Cancer. 2023;23(1). doi:10.1186/s12885-023-11364-6 PMID: 37789252

167. Kunapinun A, Songsaeng D, Buathong S, Dailey MN, Keatmanee C, Ekpanyapong M. Explainable Automated TI-RADS Evaluation of Thyroid Nodules. Sensors. 2023;23(16):7289. doi:10.3390/s23167289 PMID: 37631825

168. Liu Y, Feng Y, Qian L, Wang Z, Hu X. Deep learning diagnostic performance and visual insights in differentiating benign and malignant thyroid nodules on ultrasound images. Exp Biol Med (Maywood). 2023;248(24):2538-2546. doi:10.1177/15353702231220664 PMID: 38279511

169. Duggar WN, Vengaloor Thomas T, Wang Y, Rahman A, Wang H, Roberts PR, et al. Preoperative Prediction and Identification of Extracapsular Extension in Head and Neck Cancer Patients: Progress and Potential. Cureus. 2023. doi:10.7759/cureus.34769 PMID: 36909098

170. Yu X, Dong M, Yang D, Wang L, Wang H, Ma L. Deep learning for differentiating benign from malignant tumors on breast-specific gamma image. THC. 2023;31:61-67. doi:10.3233/thc-236007 PMID: 37038782

171. Yun J, Yun S, Park J, Cheong EN, Park S, Kim N, et al. Deep Learning of Time–Signal Intensity Curves from Dynamic Susceptibility Contrast Imaging Enables Tissue Labeling and Prediction of Survival in Glioblastoma. AJNR Am J Neuroradiol. 2023;44(5):543-552. doi:10.3174/ajnr.a7853 PMID: 37105676

172. Liu Z, Luo C, Chen X, Feng Y, Feng J, Zhang R, et al. Noninvasive prediction of perineural invasion in intrahepatic cholangiocarcinoma by clinicoradiological features and computed tomography radiomics based on interpretable machine learning: a multicenter cohort study. International Journal of Surgery. 2024;110(2):1039-1051. doi:10.1097/js9.0000000000000881 PMID: 37924497

173. Cui H, Zhao Y, Xiong S, Feng Y, Li P, Lv Y, et al. Diagnosing Solid Lesions in the Pancreas With Multimodal Artificial Intelligence. JAMA Netw Open. 2024;7(7):e2422454. doi:10.1001/jamanetworkopen.2024.22454 PMID: 39028670

174. Ye JY, Fang P, Peng ZP, Huang XT, Xie JZ, Yin XY. A radiomics-based interpretable model to predict the pathological grade of pancreatic neuroendocrine tumors. Eur Radiol. 2023;34(3):1994-2005. doi:10.1007/s00330-023-10186-1 PMID: 37658884

175. Tak D, Ye Z, Zapaischykova A, Zha Y, Boyd A, Vajapeyam S, et al. Noninvasive Molecular Subtyping of Pediatric Low-Grade Glioma with Self-Supervised Transfer Learning. Radiology: Artificial Intelligence. 2024;6(3). doi:10.1148/ryai.230333 PMID: 38446044

176. Zuo Y, Liu Q, Li N, Li P, Fang Y, Bian L, et al. Explainable 18F-FDG PET/CT radiomics model for predicting EGFR mutation status in lung adenocarcinoma: a two-center study. J Cancer Res Clin Oncol. 2024;150(10). doi:10.1007/s00432-024-05998-7 PMID: 39436414

177. Wang Y, Bai G, Liu Y, Huang M, Chen W, Wang F. Interpretable machine learning model based on CT semantic features and radiomics features to preoperatively predict Ki-67 expression in gastrointestinal stromal tumors. Sci Rep. 2024;14(1). doi:10.1038/s41598-024-80978-y PMID: 39592767

178. Duan F, Zhang M, Yang C, Wang X, Wang D. Non-invasive Prediction of Lymph Node Metastasis in NSCLC Using Clinical, Radiomics, and Deep Learning Features From 18F-FDG PET/CT Based on Interpretable Machine Learning. Academic Radiology. 2025;32(3):1645-1655. doi:10.1016/j.acra.2024.11.037 PMID: 39665892

179. Bruixola G, Dualde-Beltrán D, Jimenez-Pastor A, Nogué A, Bellvís F, Fuster-Matanzo A, et al. CT-based clinical-radiomics model to predict progression and drive clinical applicability in locally advanced head and neck cancer. Eur Radiol. 2024;35(7):4277-4288. doi:10.1007/s00330-024-11301-6 PMID: 39706922

180. Manigrasso F, Milazzo R, Russo AS, Lamberti F, Strand F, Pagnani A, et al. Mammography classification with multi-view deep learning techniques: Investigating graph and transformer-based architectures. Medical Image Analysis. 2025;99:103320. doi:10.1016/j.media.2024.103320 PMID: 39244796

181. Nakagawa J, Fujima N, Hirata K, Harada T, Wakabayashi N, Takano Y, et al. Diagnosis of skull-base invasion by nasopharyngeal tumors on CT with a deep-learning approach. Jpn J Radiol. 2024;42(5):450-459. doi:10.1007/s11604-023-01527-7 PMID: 38280100

182. Zhan Y, Song F, Zhang W, Gong T, Zhao S, Lv F. Prediction of benign and malignant pulmonary nodules using preoperative CT features: using PNI-GARS as a predictor. Front. Immunol.. 2024;15. doi:10.3389/fimmu.2024.1446511 PMID: 39635520

183. Wang H, He L, Chen X, Ding S, Xie M, Cai J. Predicting Bone Marrow Metastasis in Neuroblastoma: An Explainable Machine Learning Approach Using Contrast-Enhanced Computed Tomography Radiomics Features. Technol Cancer Res Treat. 2024;23. doi:10.1177/15330338241290386 PMID: 39440370

184. Saboor A, Li JP, Ul Haq A, Shehzad U, Khan S, Aotaibi RM, et al. DDFC: deep learning approach for deep feature extraction and classification of brain tumors using magnetic resonance imaging in E-healthcare system. Sci Rep. 2024;14(1). doi:10.1038/s41598-024-56983-6 PMID: 38494517

185. Yang Z, Lafata K, Vaios E, Hu Z, Mullikin T, Yin F, et al. Quantifying U‐Net uncertainty in multi‐parametric MRI‐based glioma segmentation by spherical image projection. Medical Physics. 2024;51(3):1931-1943. doi:10.1002/mp.16695 PMID: 37696029

186. Demirel E, Dilek O. Utilizing Radiomics of Peri‐Lesional Edema in T2‐FLAIR Subtraction Digital Images to Distinguish High‐Grade Glial Tumors From Brain Metastasis. Magnetic Resonance Imaging. 2025;61(4):1728-1737. doi:10.1002/jmri.29572 PMID: 39254002

187. Akter S, Simul Hasan Talukder M, Mondal SK, Aljaidi M, Bin Sulaiman R, Alshammari AA. Brain tumor classification utilizing pixel distribution and spatial dependencies higher-order statistical measurements through explainable ML models. Sci Rep. 2024;14(1). doi:10.1038/s41598-024-74731-8 PMID: 39468107

188. Bartnik K, Krzyziński M, Bartczak T, Korzeniowski K, Lamparski K, Wróblewski T, et al. A novel radiomics approach for predicting TACE outcomes in hepatocellular carcinoma patients using deep learning for multi-organ segmentation. Sci Rep. 2024;14(1). doi:10.1038/s41598-024-65630-z PMID: 38926517

189. Alshuhail A, Thakur A, Chandramma R, Mahesh TR, Almusharraf A, Vinoth Kumar V, et al. Refining neural network algorithms for accurate brain tumor classification in MRI imagery. BMC Med Imaging. 2024;24(1). doi:10.1186/s12880-024-01285-6 PMID: 38773391

190. Chen H, Wen Y, Wu W, Zhang Y, Pan X, Guan Y, et al. Prediction of Malignancy and Pathological Types of Solid Lung Nodules on CT Scans Using a Volumetric SWIN Transformer. J Digit Imaging. Inform. med.. 2024;38(3):1509-1517. doi:10.1007/s10278-024-01090-1 PMID: 39402355

191. Wu Y, Cao F, Lei H, Zhang S, Mei H, Ni L, et al. Interpretable multiphasic CT-based radiomic analysis for preoperatively differentiating benign and malignant solid renal tumors: a multicenter study. Abdom Radiol. 2024;49(9):3096-3106. doi:10.1007/s00261-024-04351-3 PMID: 38733392

192. Zaccaria GM, Berloco F, Buongiorno D, Brunetti A, Altini N, Bevilacqua V. A time-dependent explainable radiomic analysis from the multi-omic cohort of CPTAC-Pancreatic Ductal Adenocarcinoma. Computer Methods and Programs in Biomedicine. 2024;257:108408. doi:10.1016/j.cmpb.2024.108408 PMID: 39342876

193. M MM, T. R M, V VK, Guluwadi S. Enhancing brain tumor detection in MRI images through explainable AI using Grad-CAM with Resnet 50. BMC Med Imaging. 2024;24(1). doi:10.1186/s12880-024-01292-7 PMID: 38734629

194. Biradar S, Virupakshappa. AG-MSTLN-EL: A Multi-source Transfer Learning Approach to Brain Tumor Detection. J Digit Imaging. Inform. med.. 2024;38(1):245-261. doi:10.1007/s10278-024-01199-3 PMID: 39060764

195. Alwadee EJ, Sun X, Qin Y, Langbein FC. LATUP-Net: A lightweight 3D attention U-Net with parallel convolutions for brain tumor segmentation. Computers in Biology and Medicine. 2025;184:109353. doi:10.1016/j.compbiomed.2024.109353 PMID: 39577347

196. Haque R, Hassan MM, Bairagi AK, Shariful Islam SM. NeuroNet19: an explainable deep neural network model for the classification of brain tumors using magnetic resonance imaging data. Sci Rep. 2024;14(1). doi:10.1038/s41598-024-51867-1 PMID: 38233516

197. Wu X, Wu H, Miao S, Cao G, Su H, Pan J, et al. Deep learning prediction of esophageal squamous cell carcinoma invasion depth from arterial phase enhanced CT images: a binary classification approach. BMC Med Inform Decis Mak. 2024;24(1). doi:10.1186/s12911-023-02386-y PMID: 38167058

198. Ahmed MM, Hossain MM, Islam MR, Ali MS, Nafi AAN, Ahmed MF, et al. Brain tumor detection and classification in MRI using hybrid ViT and GRU model with explainable AI in Southern Bangladesh. Sci Rep. 2024;14(1). doi:10.1038/s41598-024-71893-3 PMID: 39354009

199. Fanizzi A, Comes MC, Bove S, Cavalera E, de Franco P, Di Rito A, et al. Explainable prediction model for the human papillomavirus status in patients with oropharyngeal squamous cell carcinoma using CNN on CT images. Sci Rep. 2024;14(1). doi:10.1038/s41598-024-65240-9 PMID: 38902523

200. Chen Y, Lin H, Sun J, Pu R, Zhou Y, Sun B. Texture Feature Differentiation of Glioblastoma and Solitary Brain Metastases Based on Tumor and Tumor-brain Interface. Academic Radiology. 2025;32(1):400-410. doi:10.1016/j.acra.2024.08.025 PMID: 39217081

201. Roy P, Srijon FMS, Bhowmik P. An explainable ensemble approach for advanced brain tumor classification applying Dual-GAN mechanism and feature extraction techniques over highly imbalanced data. PLoS ONE. 2024;19(9):e0310748. doi:10.1371/journal.pone.0310748 PMID: 39331600

202. Qu H, Chen G, Li T, Zou M, Liu J, Dong C, et al. BD-StableNet: a deep stable learning model with an automatic lesion area detection function for predicting malignancy in BI-RADS category 3–4A lesions. Phys. Med. Biol.. 2024;69(24):245002. doi:10.1088/1361-6560/ad953e PMID: 39569908

203. Gao P, Xiao Q, Tan H, Song J, Fu Y, Xu J, et al. Interpretable multi-modal artificial intelligence model for predicting gastric cancer response to neoadjuvant chemotherapy. Cell Reports Medicine. 2024;5(12):101848. doi:10.1016/j.xcrm.2024.101848 PMID: 39637859

204. Sait ARW, AlBalawi E, Nagaraj R. Ensemble learning driven Kolmogorov-Arnold Networks-based Lung Cancer classification. PLoS ONE. 2024;19(12):e0313386. doi:10.1371/journal.pone.0313386 PMID: 39739892

205. Zhao W, Hou M, Wang J, Song D, Niu Y. Interpretable machine learning model for predicting clinically significant prostate cancer: integrating intratumoral and peritumoral radiomics with clinical and metabolic features. BMC Med Imaging. 2024;24(1). doi:10.1186/s12880-024-01548-2 PMID: 39736623

206. Raptis S, Ilioudis C, Theodorou K. Uncovering the Diagnostic Power of Radiomic Feature Significance in Automated Lung Cancer Detection: An Integrative Analysis of Texture, Shape, and Intensity Contributions. BioMedInformatics. 2024;4(4):2400-2425. doi:10.3390/biomedinformatics4040129

207. Kumar K, Jyoti K, Kumar K. Machine learning for brain tumor classification: evaluating feature extraction and algorithm efficiency. Discov Artif Intell. 2024;4(1). doi:10.1007/s44163-024-00214-4

208. Spielvogel CP, Ning J, Kluge K, Haberl D, Wasinger G, Yu J, et al. Preoperative detection of extraprostatic tumor extension in patients with primary prostate cancer utilizing [68Ga]Ga-PSMA-11 PET/MRI. Insights Imaging. 2024;15(1). doi:10.1186/s13244-024-01876-5 PMID: 39666257

209. Wei L, Aryal MP, Lee C, Shah JL, Mierzwa ML, Cao Y. Interpretable survival network for progression risk analysis of multimodality imaging biomarkers in poor-prognosis head and neck cancers. Sci Rep. 2024;14(1). doi:10.1038/s41598-024-80815-2 PMID: 39622922

210. Hammad M, ElAffendi M, Asim M, Abd El-Latif AA, Hashiesh R. Automated lung cancer detection using novel genetic TPOT feature optimization with deep learning techniques. Results in Engineering. 2024;24:103448. doi:10.1016/j.rineng.2024.103448

211. Wang Q, Xiong Y, Zhu H, Mu X, Zhang Y, Ma Y. Cervical OCT image classification using contrastive masked autoencoders with Swin Transformer. Computerized Medical Imaging and Graphics. 2024;118:102469. doi:10.1016/j.compmedimag.2024.102469 PMID: 39577206

212. Gao Y, Ventura-Diaz S, Wang X, He M, Xu Z, Weir A, et al. An explainable longitudinal multi-modal fusion model for predicting neoadjuvant therapy response in women with breast cancer. Nat Commun. 2024;15(1). doi:10.1038/s41467-024-53450-8 PMID: 39511143

213. Wei Z, Bai X, Xv Y, Chen SH, Yin S, Li Y, et al. A radiomics-based interpretable machine learning model to predict the HER2 status in bladder cancer: a multicenter study. Insights Imaging. 2024;15(1). doi:10.1186/s13244-024-01840-3 PMID: 39466475

214. Liu J, Corti A, Corino VD, Mainardi L. Lung nodule classification using radiomics model trained on degraded SDCT images. Computer Methods and Programs in Biomedicine. 2024;257:108474. doi:10.1016/j.cmpb.2024.108474 PMID: 39481281

215. Muthuramalingam S, Thiyagarajan P. Impact of federated learning and explainable artificial intelligence for medical image diagnosis. IJ-AI. 2024;13(4):3772. doi:10.11591/ijai.v13.i4.pp3772-3785

216. Oladimeji OO, Ayaz H, McLoughlin I, Unnikrishnan S. Mutual information-based radiomic feature selection with SHAP explainability for breast cancer diagnosis. Results in Engineering. 2024;24:103071. doi:10.1016/j.rineng.2024.103071

217. De Benedictis SG, Gargano G, Settembre G. Enhanced MRI brain tumor detection and classification via topological data analysis and low-rank tensor decomposition. Journal of Computational Mathematics and Data Science. 2024;13:100103. doi:10.1016/j.jcmds.2024.100103

218. Jin S, Xu H, Dong Y, Wang X, Hao X, Qin F, et al. Ranking attention multiple instance learning for lymph node metastasis prediction on multicenter cervical cancer MRI. J Applied Clin Med Phys. 2024;25(12). doi:10.1002/acm2.14547 PMID: 39369718

219. Nastase INA, Moldovanu S, Biswas KC, Moraru L. Role of inter- and extra-lesion tissue, transfer learning, and fine-tuning in the robust classification of breast lesions. Sci Rep. 2024;14(1). doi:10.1038/s41598-024-74316-5 PMID: 39354128

220. Huang Z, Zhang X, Ju Y, Zhang G, Chang W, Song H, et al. Explainable breast cancer molecular expression prediction using multi-task deep-learning based on 3D whole breast ultrasound. Insights Imaging. 2024;15(1). doi:10.1186/s13244-024-01810-9 PMID: 39320560

221. Latha M, Kumar PS, Chandrika RR, Mahesh TR, Kumar VV, Guluwadi S. Revolutionizing breast ultrasound diagnostics with EfficientNet-B7 and Explainable AI. BMC Med Imaging. 2024;24(1). doi:10.1186/s12880-024-01404-3 PMID: 39223507

222. Sahlsten J, Jaskari J, Wahid KA, Ahmed S, Glerean E, He R, et al. Application of simultaneous uncertainty quantification and segmentation for oropharyngeal cancer use-case with Bayesian deep learning. Commun Med. 2024;4(1). doi:10.1038/s43856-024-00528-5 PMID: 38851837

223. Vanitha K, R MT, Sree SS, Guluwadi S. Deep learning ensemble approach with explainable AI for lung and colon cancer classification using advanced hyperparameter tuning. BMC Med Inform Decis Mak. 2024;24(1). doi:10.1186/s12911-024-02628-7 PMID: 39112991

224. Kumaran S Y, Jeya JJ, R MT, Khan SB, Alzahrani S, Alojail M. Explainable lung cancer classification with ensemble transfer learning of VGG16, Resnet50 and InceptionV3 using grad-cam. BMC Med Imaging. 2024;24(1). doi:10.1186/s12880-024-01345-x PMID: 39030496

225. Ashry ER, Maghraby FA, El-Latif YMA, Agag M. Pediatric Posterior Fossa Tumors Classification and Explanation-Driven with Explainable Artificial Intelligence Models. Int J Comput Intell Syst. 2024;17(1). doi:10.1007/s44196-024-00527-3

226. Yan L, Liang Z, Zhang H, Zhang G, Zheng W, Han C, et al. A domain knowledge-based interpretable deep learning system for improving clinical breast ultrasound diagnosis. Commun Med. 2024;4(1). doi:10.1038/s43856-024-00518-7 PMID: 38760506

227. Liu S, Amgad M, More D, Rathore MA, Salgado R, Cooper LAD. A panoptic segmentation dataset and deep-learning approach for explainable scoring of tumor-infiltrating lymphocytes. npj Breast Cancer. 2024;10(1). doi:10.1038/s41523-024-00663-1 PMID: 38942745

228. Xiang H, Xiao Y, Li F, Li C, Liu L, Deng T, et al. Development and validation of an interpretable model integrating multimodal information for improving ovarian cancer diagnosis. Nat Commun. 2024;15(1). doi:10.1038/s41467-024-46700-2 PMID: 38538600

229. Zhang X, Zhang G, Qiu X, Yin J, Tan W, Yin X, et al. Exploring non-invasive precision treatment in non-small cell lung cancer patients through deep learning radiomics across imaging features and molecular phenotypes. Biomark Res. 2024;12(1). doi:10.1186/s40364-024-00561-5 PMID: 38273398

230. Carrilero-Mardones M, Parras-Jurado M, Nogales A, Pérez-Martín J, Díez FJ. Deep Learning for Describing Breast Ultrasound Images with BI-RADS Terms. J Digit Imaging. Inform. med.. 2024;37(6):2940-2954. doi:10.1007/s10278-024-01155-1 PMID: 38926264

231. Cui K, Liu W, Wang D. Interpretable diagnosis of breast lesions in ultrasound imaging using deep multi-stage reasoning. Phys. Med. Biol.. 2024;69(21):215025. doi:10.1088/1361-6560/ad869f PMID: 39401527

232. Oumlaz M, Oumlaz Y, Oukaira A, Benelhaouare AZ, Lakhssassi A. Advancing Pulmonary Nodule Detection with ARSGNet: EfficientNet and Transformer Synergy. Electronics. 2024;13(22):4369. doi:10.3390/electronics13224369

233. Pavel MA, Islam R, Babor SB, Mehadi R, Khan R. Non-small cell lung cancer detection through knowledge distillation approach with teaching assistant. PLoS ONE. 2024;19(11):e0306441. doi:10.1371/journal.pone.0306441 PMID: 39504338

234. Talaat FM, Gamel SA, El-Balka RM, Shehata M, ZainEldin H. Grad-CAM Enabled Breast Cancer Classification with a 3D Inception-ResNet V2: Empowering Radiologists with Explainable Insights. Cancers. 2024;16(21):3668. doi:10.3390/cancers16213668 PMID: 39518105

235. Oztekin PS, Katar O, Omma T, Erel S, Tokur O, Avci D, et al. Comparison of Explainable Artificial Intelligence Model and Radiologist Review Performances to Detect Breast Cancer in 752 Patients. J of Ultrasound Medicine. 2024;43(11):2051-2068. doi:10.1002/jum.16535 PMID: 39051752

236. Hasan MZ, Hossain S, Jim RI, Bulbul AAM, Rahman MT, Moni MA. EAH-Net: A Novel Ensemble Attention-Based Hybrid Architecture for Breast Cancer Diagnosis Utilizing Ultrasound Images. Proceedings of the 1st International Workshop on Multimedia Computing for Health and Medicine. 2024:26-34. doi:10.1145/3688868.3689198

237. Lin LP, Seow ZH. Classifying Brain Tumours: A Deep Learning Approach with Explainable AI. 2024 14th International Conference on Biomedical Engineering and Technology. 2024:101-107. doi:10.1145/3678935.3678953

238. Bouamrane A, Derdour M, Alksas A, El-Baz A. Hybrid Deep Learning Approach with Feature Engineering for Enhanced Pulmonary Nodule Diagnosis. SN COMPUT. SCI.. 2024;5(7). doi:10.1007/s42979-024-03251-z

239. Liu H, She Q, Lin J, Chen Q, Fang F, Zhang Y. Attribute and Malignancy Analysis of Lung Nodule on Chest CT with Cause-and-Effect Logic. J. Med. Biol. Eng.. 2024;44(5):763-776. doi:10.1007/s40846-024-00895-3

240. Parola M, Galatolo FA, La Mantia G, Cimino MG, Campisi G, Di Fede O. Towards explainable oral cancer recognition: Screening on imperfect images via Informed Deep Learning and Case-Based Reasoning. Computerized Medical Imaging and Graphics. 2024;117:102433. doi:10.1016/j.compmedimag.2024.102433 PMID: 39276433

241. Tong G, Jiang H, Luan Q, Li X. A classification method embedding atypical patterns for distinguishing tumor subtypes in PET/CT images. Biomedical Signal Processing and Control. 2024;96:106663. doi:10.1016/j.bspc.2024.106663

242. Karunanayake N, Makhanov SS. Deep learning for ultrasound medical images: artificial life variant. Neural Comput &amp; Applic. 2024;36(28):17559-17584. doi:10.1007/s00521-024-09910-9

243. Xiong Y, Zheng Y, Long W, Wang Y, Wang Q, You Y, et al. Study on microwave ablation temperature prediction model based on grayscale ultrasound texture and machine learning. PLoS ONE. 2024;19(9):e0308968. doi:10.1371/journal.pone.0308968 PMID: 39321182

244. Tehsin S, Nasir IM, Damaševičius R, Maskeliūnas R. DaSAM: Disease and Spatial Attention Module-Based Explainable Model for Brain Tumor Detection. BDCC. 2024;8(9):97. doi:10.3390/bdcc8090097

245. Marcinkiewicz AM, Buchwald M, Shanbhag A, Bednarski BP, Killekar A, Miller RJ, et al. AI for Multistructure Incidental Findings and Mortality Prediction at Chest CT in Lung Cancer Screening. Radiology. 2024;312(3). doi:10.1148/radiol.240541 PMID: 39287522

246. Fanizzi A, Fadda F, Maddalo M, Saponaro S, Lorenzon L, Ubaldi L, et al. Developing an ensemble machine learning study: Insights from a multi-center proof-of-concept study. PLoS ONE. 2024;19(9):e0303217. doi:10.1371/journal.pone.0303217 PMID: 39255296

247. Zheng Y, Zhang Y, Lu K, Wang J, Li L, Xu D, et al. Diagnostic value of an interpretable machine learning model based on clinical ultrasound features for follicular thyroid carcinoma. Quant Imaging Med Surg. 2024;14(9):6311-6324. doi:10.21037/qims-24-601 PMID: 39281129

248. Zhao Z, Guo S, Han L, Wu L, Zhang Y, Yan B. Altruistic seagull optimization algorithm enables selection of radiomic features for predicting benign and malignant pulmonary nodules. Computers in Biology and Medicine. 2024;180:108996. doi:10.1016/j.compbiomed.2024.108996 PMID: 39137669

249. Magoulianitis V, Yang J, Yang Y, Xue J, Kaneko M, Cacciamani G, et al. PCa-RadHop: A transparent and lightweight feed-forward method for clinically significant prostate cancer segmentation. Computerized Medical Imaging and Graphics. 2024;116:102408. doi:10.1016/j.compmedimag.2024.102408 PMID: 38908295

250. Wang S, Cao C, Du T, Wang J, Li J, Li W, et al. Machine Learning Model for Predicting Axillary Lymph Node Metastasis in Clinically Node Positive Breast Cancer Based on Peritumoral Ultrasound Radiomics and SHAP Feature Analysis. J of Ultrasound Medicine. 2024;43(9):1611-1625. doi:10.1002/jum.16483 PMID: 38808580

251. Prinzi F, Orlando A, Gaglio S, Vitabile S. Breast cancer classification through multivariate radiomic time series analysis in DCE-MRI sequences. Expert Systems with Applications. 2024;249:123557. doi:10.1016/j.eswa.2024.123557

252. Carloni G, Colantonio S. Exploiting causality signals in medical images: A pilot study with empirical results. Expert Systems with Applications. 2024;249:123433. doi:10.1016/j.eswa.2024.123433

253. Koul A, Bawa RK, Kumar Y. Enhancing the detection of airway disease by applying deep learning and explainable artificial intelligence. Multimed Tools Appl. 2024;83(31):76773-76805. doi:10.1007/s11042-024-18381-y

254. Duan C, Liu Q, Wang J, Tong Q, Bai F, Han J, et al. GWO+RuleFit: rule-based explainable machine-learning combined with heuristics to predict mid-treatment FDG PET response to chemoradiation for locally advanced non-small cell lung cancer. Phys. Med. Biol.. 2024;69(15):155018. doi:10.1088/1361-6560/ad6118 PMID: 38981590

255. Meng X, Ma J, Liu F, Chen Z, Zhang T. An Interpretable Breast Ultrasound Image Classification Algorithm Based on Convolutional Neural Network and Transformer. Mathematics. 2024;12(15):2354. doi:10.3390/math12152354

256. Captier N, Orlhac F, Hovhannisyan-Baghdasarian N, Luporsi M, Girard N, Buvat I. RadShap: An Explanation Tool for Highlighting the Contributions of Multiple Regions of Interest to the Prediction of Radiomic Models. J Nucl Med. 2024;65(8):1307-1312. doi:10.2967/jnumed.124.267434 PMID: 38906555

257. Lin WC, Weng CS, Ko AT, Jan YT, Lin JB, Wu KP, et al. Interpretable machine learning model based on clinical factors for predicting muscle radiodensity loss after treatment in ovarian cancer. Support Care Cancer. 2024;32(8). doi:10.1007/s00520-024-08757-z PMID: 39046568

258. Kobayashi K, Takamizawa Y, Miyake M, Ito S, Gu L, Nakatsuka T, et al. Can physician judgment enhance model trustworthiness? A case study on predicting pathological lymph nodes in rectal cancer. Artificial Intelligence in Medicine. 2024;154:102929. doi:10.1016/j.artmed.2024.102929 PMID: 38996696

259. Yimit Y, Yasin P, Tuersun A, Wang J, Wang X, Huang C, et al. Multiparametric MRI-Based Interpretable Radiomics Machine Learning Model Differentiates Medulloblastoma and Ependymoma in Children: A Two-Center Study. Academic Radiology. 2024;31(8):3384-3396. doi:10.1016/j.acra.2024.02.040 PMID: 38508934

260. Verma S, Magazzù G, Eftekhari N, Lou T, Gilhespy A, Occhipinti A, et al. Cross-attention enables deep learning on limited omics-imaging-clinical data of 130 lung cancer patients. Cell Reports Methods. 2024;4(7):100817. doi:10.1016/j.crmeth.2024.100817 PMID: 38981473

261. Al-Tam RM, Al-Hejri AM, Alshamrani SS, Al-antari MA, Narangale SM. Multimodal breast cancer hybrid explainable computer-aided diagnosis using medical mammograms and ultrasound Images. Biocybernetics and Biomedical Engineering. 2024;44(3):731-758. doi:10.1016/j.bbe.2024.08.007

262. Ellis S, Gomes S, Trumble M, Halling-Brown MD, Young KC, Chaudhry NS, et al. Deep Learning for Breast Cancer Risk Prediction: Application to a Large Representative UK Screening Cohort. Radiology: Artificial Intelligence. 2024;6(4). doi:10.1148/ryai.230431 PMID: 38775671

263. Liu Z, Hong M, Li X, Lin L, Tan X, Liu Y. Predicting axillary lymph node metastasis in breast cancer patients: A radiomics-based multicenter approach with interpretability analysis. European Journal of Radiology. 2024;176:111522. doi:10.1016/j.ejrad.2024.111522 PMID: 38805883

264. Hermoza R, Nascimento JC, Carneiro G. Weakly-supervised preclinical tumor localization associated with survival prediction from lung cancer screening Chest X-ray images. Computerized Medical Imaging and Graphics. 2024;115:102395. doi:10.1016/j.compmedimag.2024.102395 PMID: 38729092

265. Li C, Mao Y, Liang S, Li J, Wang Y, Guo Y. Deep causal learning for pancreatic cancer segmentation in CT sequences. Neural Networks. 2024;175:106294. doi:10.1016/j.neunet.2024.106294 PMID: 38657562

266. Sung C, Oh JS, Park BS, Kim SS, Song SY, Lee JJ. Diagnostic performance of a deep-learning model using 18F-FDG PET/CT for evaluating recurrence after radiation therapy in patients with lung cancer. Ann Nucl Med. 2024;38(7):516-524. doi:10.1007/s12149-024-01925-5 PMID: 38589677

267. Li F, Zhu Tw, Lin M, Zhang Xt, Zhang Yl, Zhou Al, et al. Enhancing Ki-67 Prediction in Breast Cancer: Integrating Intratumoral and Peritumoral Radiomics From Automated Breast Ultrasound via Machine Learning. Academic Radiology. 2024;31(7):2663-2673. doi:10.1016/j.acra.2023.12.036 PMID: 38182442

268. Dudas D, Saghand PG, Dilling TJ, Perez BA, Rosenberg SA, El Naqa I. Deep Learning-Guided Dosimetry for Mitigating Local Failure of Patients With Non-Small Cell Lung Cancer Receiving Stereotactic Body Radiation Therapy. International Journal of Radiation Oncology*Biology*Physics. 2024;119(3):990-1000. doi:10.1016/j.ijrobp.2023.11.059 PMID: 38056778

269. Nowakowska S, Borkowski K, Ruppert C, Hejduk P, Ciritsis A, Landsmann A, et al. Explainable Precision Medicine in Breast MRI: A Combined Radiomics and Deep Learning Approach for the Classification of Contrast Agent Uptake. Bioengineering. 2024;11(6):556. doi:10.3390/bioengineering11060556 PMID: 38927793

270. Abdusalomov A, Rakhimov M, Karimberdiyev J, Belalova G, Cho YI. Enhancing Automated Brain Tumor Detection Accuracy Using Artificial Intelligence Approaches for Healthcare Environments. Bioengineering. 2024;11(6):627. doi:10.3390/bioengineering11060627 PMID: 38927863

271. Zhao W, Chen W, Li G, Lei D, Yang J, Chen Y, et al. GMILT: A Novel Transformer Network That Can Noninvasively Predict EGFR Mutation Status. IEEE Trans. Neural Netw. Learning Syst.. 2024;35(6):7324-7338. doi:10.1109/tnnls.2022.3190671 PMID: 35862326

272. Aziz MT, Mahmud SH, Goh KOM, Nandi D. Addressing label noise in leukemia image classification using small loss approach and pLOF with weighted-average ensemble. Egyptian Informatics Journal. 2024;26:100479. doi:10.1016/j.eij.2024.100479

273. B S, M S B. Explainable AI for Pancreatic Cancer Prediction and Survival Prognosis: An Interpretable Deep Learning and Machine Learning Approach. IJCAI. 2025;48(4). doi:10.31449/inf.v48i4.5151

274. Wehbe A, Hotiet H, Minetti I, Dellapiane S. Integrating YOLO for Advanced Content-Based Image Retrieval in Lung Cancer Imaging. 2024 31st IEEE International Conference on Electronics, Circuits and Systems (ICECS). 2024:1-4. doi:10.1109/icecs61496.2024.10848862

275. Guo M, Luo Z, Liu J, Zhou R. Mathematically-Grounded Multimodal Attention Network for Breast Cancer Prognosis. 2024 IEEE International Conference on Bioinformatics and Biomedicine (BIBM). 2024:4529-4536. doi:10.1109/bibm62325.2024.10822185

276. Mirza F, Zhao H. Hybrid Attention Mechanisms and Bio-Inspired Optimization for Enhanced Breast Cancer Diagnosis from Ultrasound Images. 2024 7th International Conference on Pattern Recognition and Artificial Intelligence (PRAI). 2024:786-792. doi:10.1109/prai62207.2024.10827528

277. Choudhury AR, Mihail RP, Chiriac SD. Using Ultra-Sound Images and a Multi-Task, Explainable Approach for Thyroid Cancer Detection. 2024 IEEE 24th International Conference on Bioinformatics and Bioengineering (BIBE). 2024:1-8. doi:10.1109/bibe63649.2024.10820466

278. Bandla Raghuramaiah, Suresh Chittineni. BreastHybridNet: A Hybrid Deep Learning Framework for Breast Cancer Diagnosis Using Mammogram Images. IJCESEN. 2025;11(1). doi:10.22399/ijcesen.812

279. Adegboro O, Ganepola V, Dietlmeier J, Mazo C, O’Connor NE. XAIMed-Net: towards explainable brain tumour detection in 2D T1-weighted CE-MRI images using transfer learning. IET Conf. Proc.. 2024;2024(10):194-201. doi:10.1049/icp.2024.3305

280. Zhang S, Liu Q. A Lung Nodule Labelling and Recognition Method Based on Variational Auto Encoder. 2024 IEEE International Conference on Medical Artificial Intelligence (MedAI). 2024:67-74. doi:10.1109/medai62885.2024.00016

281. Dugăeşescu A, Chiru CM, Nan M, Trăscău M, Florea AM. Explainable Cancer Segmentation Through Classification. 2024 IEEE 20th International Conference on Intelligent Computer Communication and Processing (ICCP). 2024:1-8. doi:10.1109/iccp63557.2024.10793024

282. Samaras AD, Feleki A, Apostolopoulos ID, Moustakidis S, Papageorgiou E, Kokkinos K, et al. Medical Decision Support System in Nuclear Medicine Diagnosis for Non-Small Cell Lung Cancer and Coronary Artery Disease: A First Stage Prototype. 2024 15th International Conference on Information, Intelligence, Systems &amp;amp; Applications (IISA). 2024:1-8. doi:10.1109/iisa62523.2024.10786612

283. Yao X, Tang M, Lu M, Zhou J, Yang D. Interpretable machine learning models for predicting skip metastasis in cN0 papillary thyroid cancer based on clinicopathological and elastography radiomics features. Front. Oncol.. 2025;14. doi:10.3389/fonc.2024.1457660 PMID: 39868368

284. Dhamdhere R, Bharadwaj S, Aggarwal A, Mutha P, Shi W, Marteau B, et al. Interpretable Survival Risk Prediction for High-Grade Glioma Patients via Radiomic Features from Peritumoral Region. 2024 46th Annual International Conference of the IEEE Engineering in Medicine and Biology Society (EMBC). 2024:1-5. doi:10.1109/embc53108.2024.10782039 PMID: 40040135

285. Shabbir A, Zubair M. Interpretable Deep Learning Classifier Using Explainable AI For Non-Small Cell Lung Cancer. 2024 Horizons of Information Technology and Engineering (HITE). 2024:1-6. doi:10.1109/hite63532.2024.10777248

286. Aly M, Ghallab A, Fathi IS. ViT-GRU: Advanced Brain Tumor Diagnosis Framework: Vision Transformer and GRU Integration for Improved MRI Analysis: A Case Study of Egypt. IEEE Access. 2024:1-1. doi:10.1109/access.2024.3513235

287. Panchawagh S. Model Agnostic Explanations to Identify Radio-Biological Associations of Radiomic Features in MRI Scans of Patients with Brain Metastases. 2024 IEEE Region 10 Symposium (TENSYMP). 2024:1-7. doi:10.1109/tensymp61132.2024.10752226

288. Parmar U, Dubey AK. Unveiling Prognostic Pattern: A Hybrid Approach For Predictive Analytics in Medical Image Data. 2024 International Conference on Electrical Electronics and Computing Technologies (ICEECT). 2024:1-6. doi:10.1109/iceect61758.2024.10739248

289. Aswin R, Arjun Kumar H, Rahul Varma U. ResNet-Based Deep Learning Framework for Liver Cancer Detection with Explainable AI (XAI) Technique. 2024 15th International Conference on Computing Communication and Networking Technologies (ICCCNT). 2024:1-6. doi:10.1109/icccnt61001.2024.10724461

290. Lamprou C, Katsikari K, Rahmani N, Hadjileontiadis LJ, Seghier M, Alshehhi A. StethoNet: Robust Breast Cancer Mammography Classification Framework. IEEE Access. 2024;12:144890-144904. doi:10.1109/access.2024.3473010

291. Sun Y, Li K, Chen D, Hu Y, Zhang S. LOMIA-T: A Transformer-Based LOngitudinal Medical Image Analysis Framework for Predicting Treatment Response of Esophageal Cancer. Lecture Notes in Computer Science. 2024:426-436. doi:10.1007/978-3-031-72086-4_40

292. Cardoso M, Santiago C, Nascimento JC. Using Counterfactual Information for Breast Classification Diagnosis. 2024 IEEE/CVF Conference on Computer Vision and Pattern Recognition Workshops (CVPRW). 2024:4996-5002. doi:10.1109/cvprw63382.2024.00505

293. Yang J, Barnett AJ, Donnelly J, Kishore S, Fang J, Schwartz FR, et al. FPN-IAIA-BL: A Multi-Scale Interpretable Deep Learning Model for Classification of Mass Margins in Digital Mammography. 2024 IEEE/CVF Conference on Computer Vision and Pattern Recognition Workshops (CVPRW). 2024:5003-5009. doi:10.1109/cvprw63382.2024.00506

294. Högberg C, Larsson S, Lång K. Engaging with artificial intelligence in mammography screening: Swedish breast radiologists’ views on trust, information and expertise. DIGITAL HEALTH. 2024;10. doi:10.1177/20552076241287958 PMID: 39381821

295. Bhattacharya S, Saleem SM, Singh A, Singh S, Tripathi S. Empowering precision medicine: regenerative AI in breast cancer. Front. Oncol.. 2024;14. doi:10.3389/fonc.2024.1465720 PMID: 39372870

296. Manuela UM, Nakasi R, Jjingo D, Hellen N, Ngobye M, Marvin G. Machine Vision Intelligence Using Layer-Wise Relevance Backward Propagation For Breast Cancer Diagnosis. 2024 5th International Conference on Image Processing and Capsule Networks (ICIPCN). 2024:143-149. doi:10.1109/icipcn63822.2024.00031

297. Burgos D, Morshed A, Rashid MM, Mandala S. A Comparison of Machine Learning Models to Deep Learning Models for Cancer Image Classification and Explainability of Classification. 2024 International Conference on Data Science and Its Applications (ICoDSA). 2024:386-390. doi:10.1109/icodsa62899.2024.10651790

298. Chakravarthy S, Nagarajan B, Khan SB, Venkatesan VK, Ramakrishna MT, Musharraf AA, et al. Spatial Attention Integrated EfficientNet Architecture for Breast Cancer Classification with Explainable AI. CMC. 2024;80(3):5029-5045. doi:10.32604/cmc.2024.052531

299. Zhang Y, Zeng B, Li J, Zheng Y, Chen X. A Multi-Task Transformer With Local-Global Feature Interaction and Multiple Tumoral Region Guidance for Breast Cancer Diagnosis. IEEE J. Biomed. Health Inform.. 2024;28(11):6840-6853. doi:10.1109/jbhi.2024.3454000 PMID: 39226204

300. Ma X, Ning F, Xu X, Shan J, Li H, Tian X, et al. Survival Prediction for Non-Small Cell Lung Cancer Based on Multimodal Fusion and Deep Learning. IEEE Access. 2024;12:123236-123249. doi:10.1109/access.2024.3453930

301. Harris C, Okorie U, Makrogiannis S. Mammographic Breast Density Classification by Integration of Deep Dictionaries and Multi-Model Sparse Approximations. 2024 IEEE International Symposium on Biomedical Imaging (ISBI). 2024:1-5. doi:10.1109/isbi56570.2024.10635360

302. Yang F, Jemaa S, Bengtsson T, Ghaoui LE. Metastatic Lung Cancer Prognosis Via Deep Image-Based Lesion Prioritization. 2024 IEEE International Symposium on Biomedical Imaging (ISBI). 2024:1-5. doi:10.1109/isbi56570.2024.10635163

303. Li S, Liu B, Deng F, Xu Y, Zhou W. Image Synthesis of Hepatobiliary Phase using Contrast-Enhanced MRI and Diffusion Model. 2024 IEEE International Symposium on Biomedical Imaging (ISBI). 2024:1-5. doi:10.1109/isbi56570.2024.10635567

304. Mary AA, Thanammal KK. BlockChain and Deep Learning with Dynamic Pattern Features for Lung Cancer Diagnosis. ijacsa. 2024;15(8). doi:10.14569/ijacsa.2024.01508106

305. Zhang R, Zhu H, Chen M, Sang W, Lu K, Li Z, et al. A dual-radiomics model for overall survival prediction in early-stage NSCLC patient using pre-treatment CT images. Front. Oncol.. 2024;14. doi:10.3389/fonc.2024.1419621 PMID: 39206157

306. Muftah A, Shermer SM, Langbein FC. Texture Feature Analysis for Classification of Early-Stage Prostate Cancer in MpMRI. Lecture Notes in Computer Science. 2024:118-131. doi:10.1007/978-3-031-67285-9_9

307. Yao S, Shen P, Dai F, Deng L, Qiu X, Zhao Y, et al. Thyroid Cancer Central Lymph Node Metastasis Risk Stratification Based on Homogeneous Positioning Deep Learning. Research. 2024;7. doi:10.34133/research.0432 PMID: 39165637

308. Li J. Deep Learning Modeling and Increasing Interpretability of Lung Nodule Classification. 2024 16th International Conference on Electronics, Computers and Artificial Intelligence (ECAI). 2024:1-7. doi:10.1109/ecai61503.2024.10607434

309. G P, L A. Novel AI Multimodal Approach for Combating Against Pulmonary Carcinoma. 2024 5th International Conference for Emerging Technology (INCET). 2024:1-6. doi:10.1109/incet61516.2024.10593234

310. Pathak S, Schlötterer J, Veltman J, Geerdink J, van Keulen M, Seifert C. Prototype-Based Interpretable Breast Cancer Prediction Models: Analysis and Challenges. Communications in Computer and Information Science. 2024:21-42. doi:10.1007/978-3-031-63787-2_2

311. Fernandes L, Pereira T, Oliveira HP. Exploring the differences between Multi-task and Single-task with the use of Explainable AI for lung nodule classification. 2024 IEEE 37th International Symposium on Computer-Based Medical Systems (CBMS). 2024:418-423. doi:10.1109/cbms61543.2024.00075

312. Shariaty F, Pavlov VA, Fedyashina SV, Serebrennikov NA. Integrating Deep Learning and Explainable AI for Non-Invasive Prediction of EGFR and KRAS Mutations in NSCLC: A Novel Radiogenomic Approach. 2024 V International Conference on Neural Networks and Neurotechnologies (NeuroNT). 2024:32-35. doi:10.1109/neuront62606.2024.10585441

313. Ramasamy P, Alabdulkreem E, Alruwais N, Gladis Pushparathi VP. An attention-based neural network for lung cancer classification and gradient in MRI. Automatika. 2024;65(4):1379-1390. doi:10.1080/00051144.2024.2376776

314. Rifi AL, Raets C, Dufait I, De Ridder M, Barbé K. Integrating radiomics and immunology: non-invasive assessment of CD8+ T cell levels. 2024 IEEE International Instrumentation and Measurement Technology Conference (I2MTC). 2024:1-4. doi:10.1109/i2mtc60896.2024.10560686

315. Ruan Y, Jin R, Liu Z, Wang C, Zhang L, Peng T. Delineation of Prostate Cancer Via Enhanced AI-Based Algorithm In Ultrasound Images. ICASSP 2024 - 2024 IEEE International Conference on Acoustics, Speech and Signal Processing (ICASSP). 2024:2275-2279. doi:10.1109/icassp48485.2024.10446346

316. Kumar KN, V C SS, Mane A, K R, Nirmal G, Vaghulade M. Enhancing Lung Cancer Detection and Localization with a Hybrid VGG-19 and Adaptive Neuro-Fuzzy Inference System (ANFIS) Approach on Imaging Data. 2024 Systems and Information Engineering Design Symposium (SIEDS). 2024:280-285. doi:10.1109/sieds61124.2024.10534680

317. Yang X, Gao C, Sun N, Qin X, Liu X, Zhang C. An interpretable clinical ultrasound-radiomics combined model for diagnosis of stage I cervical cancer. Front. Oncol.. 2024;14. doi:10.3389/fonc.2024.1353780 PMID: 38846980

318. Muntasir F, Datta A, Mahmud S. Interpreting Multiclass Lung Cancer from CT Scans using Grad-CAM on Lightweight CNN Layers. 2024 6th International Conference on Electrical Engineering and Information &amp;amp; Communication Technology (ICEEICT). 2024:208-213. doi:10.1109/iceeict62016.2024.10534491

319. Ahmed ST, Barua S, Fahim-Ul-Islam M, Chakrabarty A. CoAtNet-Lite: Advancing Mammogram Mass Detection Through Lightweight CNN - Transformer Fusion with Attention Mapping. 2024 6th International Conference on Electrical Engineering and Information &amp;amp; Communication Technology (ICEEICT). 2024:143-148. doi:10.1109/iceeict62016.2024.10534420

320. Kuang B, Zhang J, Zhang M, Xia H, Qiang G, Zhang J. Advancing NSCLC pathological subtype prediction with interpretable machine learning: a comprehensive radiomics-based approach. Front. Med.. 2024;11. doi:10.3389/fmed.2024.1413990 PMID: 38841579

321. Wedisinghe H, Fernando T. Explainable AI for Early Lung Cancer Detection: A Path to Confidence. 2024 4th International Conference on Advanced Research in Computing (ICARC). 2024:13-18. doi:10.1109/icarc61713.2024.10499787

322. Dharani M, Radhakrishnan C. Optimizing Breast Cancer Prediction: A Multimodal Dataset Apporach with XGBOOST. 2024 International Conference on Emerging Smart Computing and Informatics (ESCI). 2024:1-8. doi:10.1109/esci59607.2024.10497437

323. Hasan Y, Lima Ad, Amerehi F, Bulnes DRFd, Healy P, Ryan C. Interpretable Solutions for Breast Cancer Diagnosis with Grammatical Evolution and Data Augmentation. Lecture Notes in Computer Science. 2024:224-239. doi:10.1007/978-3-031-56852-7_15

324. Enhancing Brain Tumor Detection and Classification with Reduced Complexity Spatial Fusion Convolutional Neural Networks. IJIES. 2024;17(1):263-277. doi:10.22266/ijies2024.0229.25

325. Nicoletti G, Mazzetti S, Maimone G, Cignini V, Cuocolo R, Faletti R, et al. Development and Validation of an Explainable Radiomics Model to Predict High-Aggressive Prostate Cancer: A Multicenter Radiomics Study Based on Biparametric MRI. Cancers. 2024;16(1):203. doi:10.3390/cancers16010203 PMID: 38201630

326. Lv T, Hong X, Liu Y, Miao K, Sun H, Li L, et al. AI-powered interpretable imaging phenotypes noninvasively characterize tumor microenvironment associated with diverse molecular signatures and survival in breast cancer. Computer Methods and Programs in Biomedicine. 2024;243:107857. doi:10.1016/j.cmpb.2023.107857 PMID: 37865058

327. Wang C, Chen Y, Liu F, Elliott M, Kwok CF, Peña-Solorzano C, et al. An Interpretable and Accurate Deep-Learning Diagnosis Framework Modeled With Fully and Semi-Supervised Reciprocal Learning. IEEE Trans. Med. Imaging. 2024;43(1):392-404. doi:10.1109/tmi.2023.3306781 PMID: 37603481

328. Zhou S, Islam UJ, Pfeiffer N, Banerjee I, Patel BK, Iquebal AS. SCGAN: Sparse CounterGAN for Counterfactual Explanations in Breast Cancer Prediction. IEEE Trans. Automat. Sci. Eng.. 2024;21(3):2264-2275. doi:10.1109/tase.2023.3333788 PMID: 40734689

329. Ahmed M, Bibi T, Khan RA, Nasir S. Enhancing Breast Cancer Diagnosis in Mammography: Evaluation and Integration of Convolutional Neural Networks and Explainable AI. 2024 26th International Multi-Topic Conference (INMIC). 2024:1-6. doi:10.1109/inmic64792.2024.11004362

330. Hasan SA, Mahim SM, Hossen ME, Hasan MO, Ashik TH, Ahmmed F, et al. DSP-UNet: Dual-Skip Perceiver UNet for Lower-Grade Glioma Segmentation. 2024 27th International Conference on Computer and Information Technology (ICCIT). 2024:3069-3074. doi:10.1109/iccit64611.2024.11022458

331. Khandaker MAA, Raha ZS, Iqbal SB, Mridha M, Shin J. From Images to Insights: Transforming Brain Cancer Diagnosis with Explainable AI. 2024 27th International Conference on Computer and Information Technology (ICCIT). 2024:891-896. doi:10.1109/iccit64611.2024.11022512

332. Fahim Faiyaz GM, Abbas Uddin Tasin M, Azhar T, Uddin MN. A Hybrid Deep Learning Approach For Brain Tumor Detection Using XAI with GradCAM. 2024 27th International Conference on Computer and Information Technology (ICCIT). 2024:1235-1240. doi:10.1109/iccit64611.2024.11021963

333. Kaushik S, Lamba AK, Kansal I, Khullar V, Sharma P. Explainable Deep Learning for Lung Cancer Detection: Comparing CNN and DenseNet201 with Grad-CAM. 2024 2nd International Conference on Signal Processing, Communication, Power and Embedded System (SCOPES). 2024:1-5. doi:10.1109/scopes64467.2024.10990463

334. Gao T, Song J, Yu X, Zhang S, Liang W, Zhang H, et al. Loose Lesion Location Self-supervision Enhanced Colorectal Cancer Diagnosis. Lecture Notes in Computer Science. 2024:415-425. doi:10.1007/978-3-031-72120-5_39

335. Bunnell A, Glaser Y, Valdez D, Wolfgruber T, Altamirano A, Zamora González C, et al. Learning a Clinically-Relevant Concept Bottleneck for Lesion Detection in Breast Ultrasound. Lecture Notes in Computer Science. 2024:650-659. doi:10.1007/978-3-031-72384-1_61

336. Sarker S. Transfer Learning and Explainable AI for Brain Tumor Classification: A Study Using MRI Data from Bangladesh. 2024 6th International Conference on Sustainable Technologies for Industry 5.0 (STI). 2024:1-6. doi:10.1109/sti64222.2024.10951092

337. Sharma J, Kumar D, Malhotra A. A Swift and Accurate Approach to Breast Cancer Recognition Using CNN and Decision Tree Method. 2024 5th IEEE Global Conference for Advancement in Technology (GCAT). 2024:1-5. doi:10.1109/gcat62922.2024.10924071

338. Chang SC, Yu HP, Hsieh YH, Wang P, Wang W, Su TH, et al. ReViT: A Hybrid Approach for BCLC Staging of Hepatocellular Carcinoma Using 3D CT with Multiple Instance Learning. 2024 IEEE EMBS International Conference on Biomedical and Health Informatics (BHI). 2024:1-8. doi:10.1109/bhi62660.2024.10913759

339. Hassan MR, Hassan MM, Rahman MA. Comparative Analysis of LIME and Shape Analysis Techniques in Prostate Cancer MRI Interpretation. 2024 International Conference on Engineering and Emerging Technologies (ICEET). 2024:1-6. doi:10.1109/iceet65156.2024.10913801

340. Ye Z, Ge S, Yang M, Du C, Ma F. An Explainable Classification Model of Renal Cancer Subtype Using Deep Learning. 2024 17th International Congress on Image and Signal Processing, BioMedical Engineering and Informatics (CISP-BMEI). 2024:1-10. doi:10.1109/cisp-bmei64163.2024.10906194

341. Ariyametkul A, Tamang S, Paing MP. Explainable AI (XAI) for Breast Cancer Diagnosis. 2024 16th Biomedical Engineering International Conference (BMEiCON). 2024:1-5. doi:10.1109/bmeicon64021.2024.10896324

342. Prinzi F, Orlando A, Gaglio S, Vitabile S. Interpretable Radiomic Signature for Breast Microcalcification Detection and Classification. J Digit Imaging. Inform. med.. 2024;37(3):1038-1053. doi:10.1007/s10278-024-01012-1 PMID: 38351223

343. Li G, Huang Q, Liu C, Wang G, Guo L, Liu R, et al. Fully automated diagnosis of thyroid nodule ultrasound using brain-inspired inference. Neurocomputing. 2024;582:127497. doi:10.1016/j.neucom.2024.127497

344. M S, K S D, D M, V R. A Deep Learning Approach for Efficient Breast Cancer Diagnosis Using Hybrid CNN-BILSTM with Soft Attention Mechanism. SSRG-IJECE. 2024;11(5):130-138. doi:10.14445/23488549/ijece-v11i5p114

345. Qiu J, Mitra J, Ghose S, Dumas C, Yang J, Sarachan B, et al. A Multichannel CT and Radiomics-Guided CNN-ViT (RadCT-CNNViT) Ensemble Network for Diagnosis of Pulmonary Sarcoidosis. Diagnostics. 2024;14(10):1049. doi:10.3390/diagnostics14101049 PMID: 38786347

346. Huang Y, Wang X, Cao Y, Li M, Li L, Chen H, et al. Multiparametric MRI model to predict molecular subtypes of breast cancer using Shapley additive explanations interpretability analysis. Diagnostic and Interventional Imaging. 2024;105(5):191-205. doi:10.1016/j.diii.2024.01.004 PMID: 38272773

347. Zhang Y, Yang Y, Ma Y, Liu Y, Ye Z. Development and validation of an interpretable radiomic signature for preoperative estimation of tumor mutational burden in lung adenocarcinoma. Front. Genet.. 2024;15. doi:10.3389/fgene.2024.1367434 PMID: 38660677

348. Comes MC, Fanizzi A, Bove S, Didonna V, Diotiaiuti S, Fadda F, et al. Explainable 3D CNN based on baseline breast DCE-MRI to give an early prediction of pathological complete response to neoadjuvant chemotherapy. Computers in Biology and Medicine. 2024;172:108132. doi:10.1016/j.compbiomed.2024.108132 PMID: 38508058

349. Cerekci E, Alis D, Denizoglu N, Camurdan O, Ege Seker M, Ozer C, et al. Quantitative evaluation of Saliency-Based Explainable artificial intelligence (XAI) methods in Deep Learning-Based mammogram analysis. European Journal of Radiology. 2024;173:111356. doi:10.1016/j.ejrad.2024.111356 PMID: 38364587

350. Yang L, Shao D, Huang Z, Geng M, Zhang N, Chen L, et al. Few‐shot segmentation framework for lung nodules via an optimized active contour model. Medical Physics. 2024;51(4):2788-2805. doi:10.1002/mp.16933 PMID: 38189528

351. Belue MJ, Law YM, Marko J, Turkbey E, Malayeri A, Yilmaz EC, et al. Deep Learning-Based Interpretable AI for Prostate T2W MRI Quality Evaluation. Academic Radiology. 2024;31(4):1429-1437. doi:10.1016/j.acra.2023.09.030 PMID: 37858505

352. Peng T, Wu Y, Zhao J, Wang C, Jackie Wu Q, Cai J. Organ boundary delineation for automated diagnosis from multi-center using ultrasound images. Expert Systems with Applications. 2024;238:122128. doi:10.1016/j.eswa.2023.122128

353. Donnelly J, Moffett L, Barnett AJ, Trivedi H, Schwartz F, Lo J, et al. AsymMirai: Interpretable Mammography-based Deep Learning Model for 1–5-year Breast Cancer Risk Prediction. Radiology. 2024;310(3). doi:10.1148/radiol.232780 PMID: 38501952

354. Zhao Q, Chang C, Yang X, Zhao L. Robust explanation supervision for false positive reduction in pulmonary nodule detection. Medical Physics. 2024;51(3):1687-1701. doi:10.1002/mp.16937 PMID: 38224306

355. Tang L, Zhang Z, Yang J, Feng Y, Sun S, Liu B, et al. A New Automated Prognostic Prediction Method Based on Multi-Sequence Magnetic Resonance Imaging for Hepatic Resection of Colorectal Cancer Liver Metastases. IEEE J. Biomed. Health Inform.. 2024;28(3):1528-1539. doi:10.1109/jbhi.2024.3350247 PMID: 38446655

356. Wang Y, Rahman A, Duggar WN, Thomas TV, Roberts PR, Vijayakumar S, et al. A gradient mapping guided explainable deep neural network for extracapsular extension identification in 3D head and neck cancer computed tomography images. Medical Physics. 2024;51(3):2007-2019. doi:10.1002/mp.16680 PMID: 37643447

357. Wang Z, Zhang Z, Traverso A, Dekker A, Qian L, Sun P. Assessing the role of GPT-4 in thyroid ultrasound diagnosis and treatment recommendations: enhancing interpretability with a chain of thought approach. Quant Imaging Med Surg. 2024;14(2):1602-1615. doi:10.21037/qims-23-1180 PMID: 38415150

358. Wang J, Dai G, Ren X, Shi R, Luo R, Liu J, et al. Assessing the reproducibility, stability, and biological interpretability of multimodal computed tomography image features for prognosis in advanced non‐small cell lung cancer. iRADIOLOGY. 2024;2(1):3-16. doi:10.1002/ird3.56

359. Islam MK, Rahman MM, Ali MS, Mahim S, Miah MS. Enhancing lung abnormalities diagnosis using hybrid DCNN-ViT-GRU model with explainable AI: A deep learning approach. Image and Vision Computing. 2024;142:104918. doi:10.1016/j.imavis.2024.104918

360. Song X, Duan X, He X, Wang Y, Li K, Deng B, et al. Computer-aided diagnosis of distal metastasis in non-small cell lung cancer by low-dose CT based radiomics and deep learning signatures. Radiol med. 2024;129(2):239-251. doi:10.1007/s11547-024-01770-6 PMID: 38214839

361. Lanjewar MG, Panchbhai KG, Patle LB. Fusion of transfer learning models with LSTM for detection of breast cancer using ultrasound images. Computers in Biology and Medicine. 2024;169:107914. doi:10.1016/j.compbiomed.2023.107914 PMID: 38190766

362. Wang C, Liu Y, Wang F, Zhang C, Wang Y, Yuan M, et al. Towards reliable and explainable AI model for pulmonary nodule diagnosis. Biomedical Signal Processing and Control. 2024;88:105646. doi:10.1016/j.bspc.2023.105646

363. Tasnim J, Hasan MK. CAM-QUS guided self-tuning modular CNNs with multi-loss functions for fully automated breast lesion classification in ultrasound images. Phys. Med. Biol.. 2024;69(1):015018. doi:10.1088/1361-6560/ad1319 PMID: 38056017

364. Liu W, Zhou M, Mo H, Zhao S. A Multimodal Breast Cancer Diagnosis Approach Based on EfficientNet and Hybrid Feature Selection. 2024 China Automation Congress (CAC). 2024:7339-7343. doi:10.1109/cac63892.2024.10865739

365. Srivastava P, Mishra AR, Chauhan SS. Enhancing Robustness of Deep Learning Model for Lung Cancer Detection: Adversarial Training and Gradient-Based Interpretability. 2024 1st International Conference on Sustainable Computing and Integrated Communication in Changing Landscape of AI (ICSCAI). 2024:1-5. doi:10.1109/icscai61790.2024.10866616

366. Butt AUR, Asif M, Rashid T, Ali H, Raoof S, Raoof F. Beyond Boundaries: A Novel Ensemble Approach for Breast Cancer Detection in Ultrasound Imaging Using Deep Learning. 2024 21st International Bhurban Conference on Applied Sciences and Technology (IBCAST). 2024:315-320. doi:10.1109/ibcast61650.2024.10877027

367. Ali Shah SM, Jamil D, Ali Khan MN, Mohammed Al-Jarwani FM. Predicting Lung Cancer Risk Using Artificial Intelligence. 2024 2nd International Conference on Computing and Data Analytics (ICCDA). 2024:1-4. doi:10.1109/iccda64887.2024.10867365

368. V VP, Chattu P, Sivasankari K, Pisal DT, Renuka Sai B, Suganthi D. Exploring Convolution Neural Networks for Image Classification in Medical Imaging. 2024 International Conference on Intelligent and Innovative Technologies in Computing, Electrical and Electronics (IITCEE). 2024:1-4. doi:10.1109/iitcee59897.2024.10467794

369. Surkov YI, Serebryakova IA, Kuzinova YK, Konopatskova OM, Safronov DV, Kapralov SV, et al. Multimodal Method for Differentiating Various Clinical Forms of Basal Cell Carcinoma and Benign Neoplasms In Vivo. Diagnostics. 2024;14(2):202. doi:10.3390/diagnostics14020202 PMID: 38248078

370. Panda NR, Muduli D, Sharma SK. Customized MobileNet with Transfer Learning for Enhanced Early Breast Cancer Detection: A Deep Learning Approach. 2024 2nd International Conference on Signal Processing, Communication, Power and Embedded System (SCOPES). 2024:1-5. doi:10.1109/scopes64467.2024.10991324

371. Dhiman B, Kamboj S, Srivastava V. Explainable AI based efficient ensemble model for breast cancer classification using optical coherence tomography. Biomedical Signal Processing and Control. 2024;91:106007. doi:10.1016/j.bspc.2024.106007
