# Supplementary material for: Explainable AI in Cancer Imaging: Scoping Review of Methods, Modalities, and Clinical Integration
Source: J Med Internet Res. 2026 May 20;28:e80645. doi: 10.2196/80645 (PMC13189567; doi:10.2196/80645)
Supplement: Multimedia Appendix 4 [file jmir-v28-e80645-s004.docx]

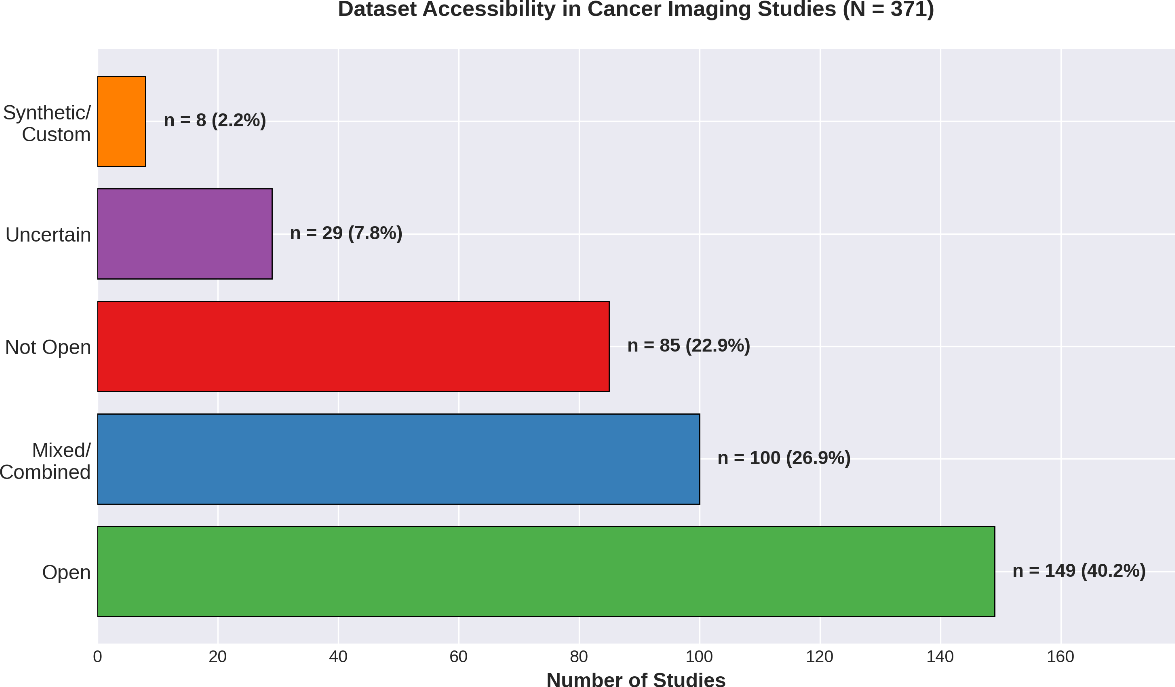


**Figure S1**. Accessibility of datasets used in studies applying artificial intelligence to cancer imaging (2017-2024; N=371). Studies were classified as open (40.2%), mixed/combined (26.9%), not open (22.9%), uncertain (7.8%), or synthetic/custom (2.2%). The corpus includes major cancers (breast, lung, brain, liver, prostate) from multiple continents.


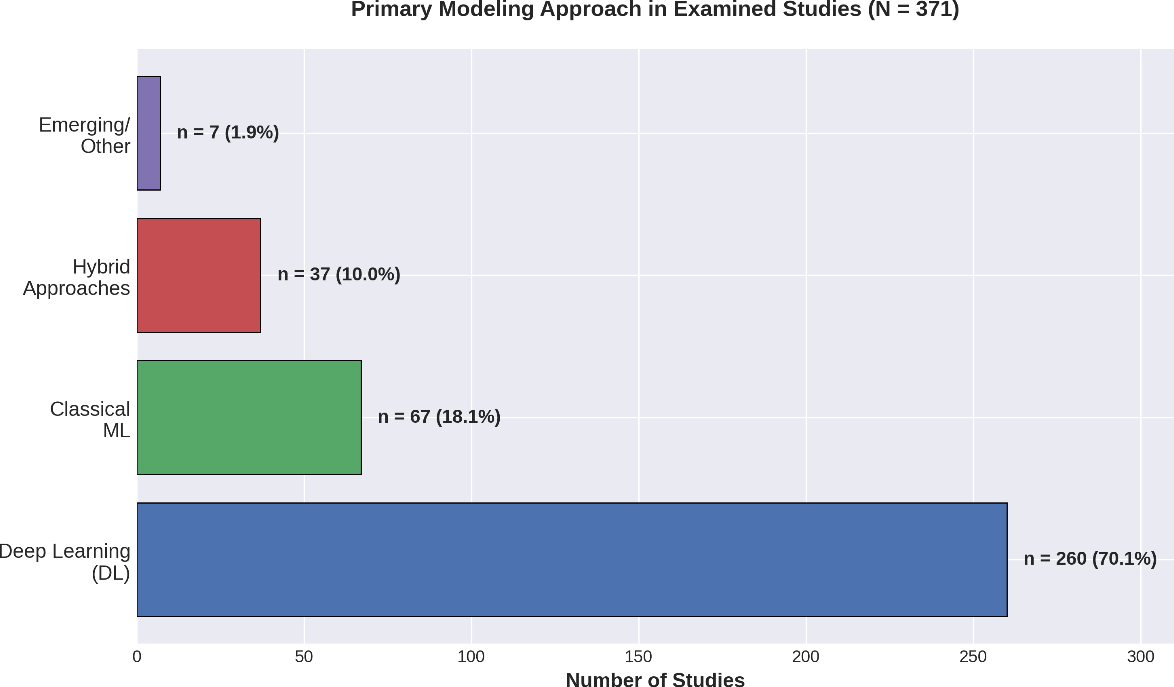


**Figure S2.** Distribution of primary modeling approaches among examined studies (2017-2024; n=371). Deep learning architectures (n=260, 70.1%) were predominant, followed by classical Machine learning (n=67, 18.1%), hybrid pipelines (n=37, 10.0%), and emerging paradigms (n=7, 1.9%).


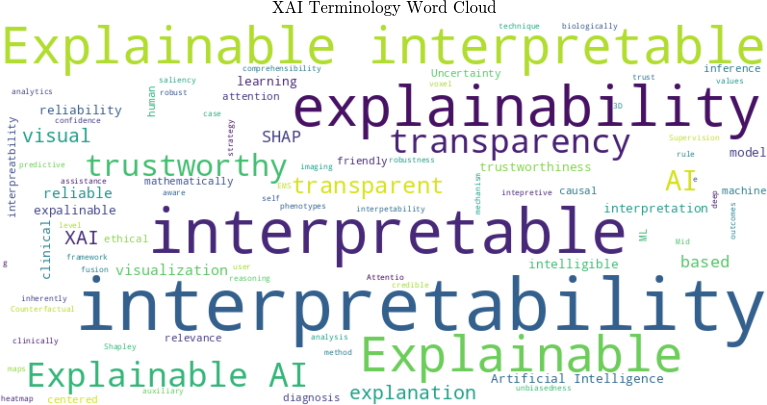


**Figure S3.** Word cloud of explainable artificial intelligence terminology in the reviewed literature (2017-2024). Illustrative overview of terminology appearing in the reviewed corpus. The terminology reflects the authors’ own descriptors for their xAI components/solutions. Word size reflects frequency of appearance in the study but should be interpreted qualitatively. Because explainable and interpretable were seed search terms, their prominence reflects both query design and common usage.


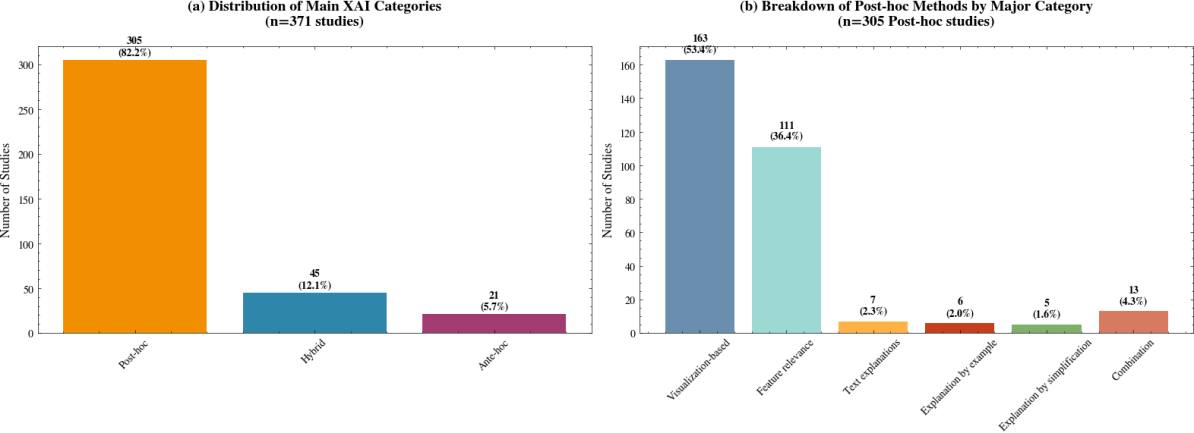


**Figure S4.** Distribution of explainable artificial intelligence (xAI) methods across included studies (n=371).
